# Assessment of the Potential of *Sarcandra glabra (Thunb.)* Nakai. in Treating Ethanol-Induced Gastric Ulcer in Rats Based on Metabolomics and Network Analysis

**DOI:** 10.3389/fphar.2022.810344

**Published:** 2022-07-12

**Authors:** Chao Li, Rou Wen, DeWen Liu, LiPing Yan, Qianfeng Gong, Huan Yu

**Affiliations:** ^1^ School of Pharmacy, Tianjin University of Traditional Chinese Medicine, Tianjin, China; ^2^ School of Pharmacy, Jiangxi University of Chinese Medicine, Nanchang, China; ^3^ Institute of Chinese Materia Medica, China Academy of Chinese Medical Sciences, Beijing, China

**Keywords:** metabolomics, network pharmacology, *Sarcandra glabra* (thunb.) nakai, gastric ulcer, gastroprotection

## Abstract

Gastric ulcer (GU) is one of the most commonly diagnosed diseases worldwide, threatening human health and seriously affecting quality of life. Reports have shown that the Chinese herbal medicine *Sarcandra glabra* (Thunb.) Nakai (SGN) can treat GU. However, its pharmacological effects deserve further validation; in addition, its mechanism of action is unclear. An acute gastric ulcer (AGU) rat model induced by alcohol was used to evaluate the gastroprotective effect of SGN by analysis of the histopathological changes in stomach tissue and related cytokine levels; the potential mechanisms of action of SGN were investigated via serum metabolomics and network pharmacology. Differential metabolites of rat serum were identified by metabolomics and the metabolic pathways of the identified metabolites were enriched via MetaboAnalyst. Furthermore, the critical ingredients and candidate targets of SGN anti-AGU were elucidated. A compound-reaction-enzyme-gene network was established using Cytoscape version 3.8.2 based on integrated analysis of metabolomics and network pharmacology. Finally, molecular docking was applied to verify the acquired key targets. The results showed that SGN exerted a certain gastroprotective effect via multiple pathways and targets. The effects of SGN were mainly caused by the key active ingredients isofraxidin, rosmarinic, and caffeic acid, which regulate hub targets, such as PTGS2, MAPK1, and KDR, which maintain the homeostasis of related metabolites. Signal pathways involved energy metabolism as well as immune and amino acid metabolism. Overall, the multi-omics techniques were proven to be promising tools in illuminating the mechanism of action of SGN in protecting against diseases. This integrated strategy provides a basis for further research and clinical application of SGN.

## Graphical Abstract

Flowchart of multi-omics integrated analysis. The part 1: The mechanism of action of SGN against AGU was comprehensively clarified based on non-targeted metabolomics. The part 2: The active ingredients anti-AGU in SGN, the potential hub genes and their protective mechanisms were identified by performing network pharmacology. Part 3: The potential connection between biomarkers and hub genes was obtained and molecular docking was applied to verify the acquired key targets with the key ingredient.
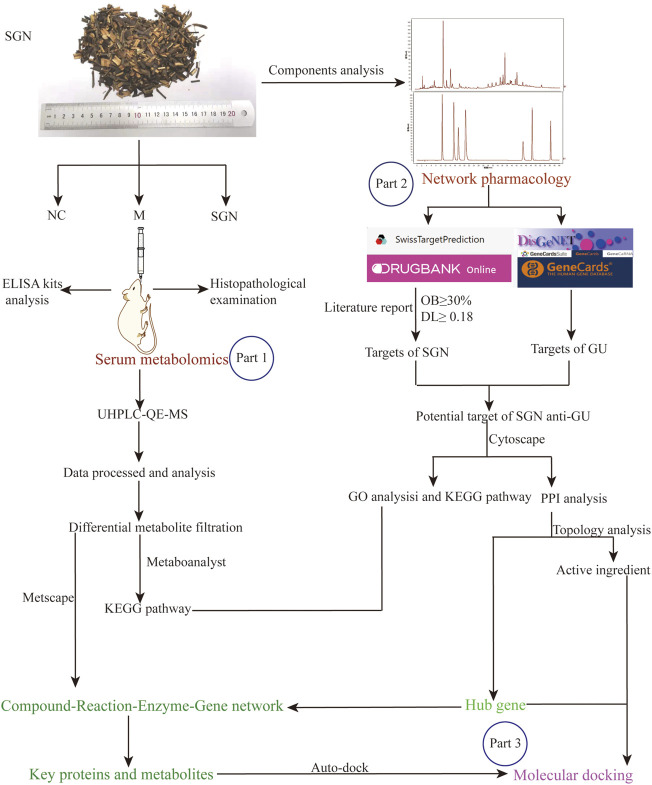

## 1 Introduction

Gastric ulcer (GU) is a common peptic ulcer caused by the destruction of the gastric mucosa. GU is hard to cure and can easily induce infection; GU relapse is common. Based on epidemiological surveys, the incidence of GU in Western countries about 2.4%. Furthermore, the incidence of GU in some areas of mainland China is up to about 6.07% and approximately 22.5% of patients with gastrointestinal symptoms have GU (Li et al., 2010). Besides impacting human health, GU also has a heavy economic burden on patients and their families. For instance, the average annual medical cost of treating GU patients in the United States is 23,819 United States dollars and 59.6–2,553.10 United States dollars in South Korea (Song et al., 2013). GU has a complex pathogenesis that is unclear and can be caused by a variety of factors, such as alcohol, drugs, and *Helicobacter pylori*; however, it is mainly caused by the imbalance of aggressive factors and defense mechanisms of the gastric mucosa (Valcheva-Kuzmanova et al., 2019). It is reported that alcohol is one of the typical gastric mucosal attack factors. Alcohol is first digested and absorbed by the digestive system, including the gastrointestinal tract, before entering the blood; therefore, intake of highly concentrated alcohol erodes, destroys the protective layer, and causes irreversible damage to the gastric mucosa through the digestion of gastric mucus and bicarbonate, thus inducing acute gastric ulcers (AGU) (Zheng et al., 2016; Zhang et al., 2019). Additionally, the histological characteristics and healing process of the rat alcoholic GU model are similar to those of human gastric mucosal damage; it can be produced efficiently and can effectively model similar systems. This model has been widely used in research regarding gastroprotective medicine (Bai et al., 2020; Lian et al., 2020). Pathological studies have shown that the onset of acute alcoholic GU is closely associated with neutrophil infiltration, release of pro-inflammatory factors, and oxidative stress (Zheng et al., 2016). In recent decades, various specific treatments for GU have been found, with drug therapy being the primary GU treatment method. Appropriate medication regiments, such as proton pump inhibitors (PPIs), *Helicobacter pylori* eradication, and nonsteroidal anti-inflammatory drugs (NSAIDs), can effectively treat GU patients. However, several reports have shown that these drugs have some safety issues and side effects after long-term chronic treatment. Some of the safety issues are related to the possible long-term effects of chronic hypergastrinemia (Lee et al., 2019), leading to loss of muscle function (Vinke et al., 2020), nephrotic syndrome, and chronic renal failure (Harirforoosh and Jamali, 2009).

Therefore, more effective and less toxic therapies are needed for GU treatment. Both clinical and experimental studies have demonstrated that herbal medicines exert protective effects against GU with fewer side effects and lower medical expenses (Devanesan et al., 2018; Zhang et al., 2018; Sudi et al., 2020). Traditional Chinese Medicine (TCM) has been widely used in China for maintaining health and treating diseases for several years. *Sarcandra glabra* (thunb.) Nakai (SGN) (Chinese name called Zhong jie feng, Jiu jie cha, or cao shan hu) is an essential herb used in TCM with a range of biological applications, including treating cancer, rheumatism, pneumonia, digestive tract inflammations, traumatic injuries, and fractures (Zhou et al., 2013). Furthermore, SGN has been widely used in food, medicine, health care products, cosmetics, etc., It has been demonstrated that SGN has great development potential. In addition, several studies have demonstrated that SGN exhibits anti-inflammatory activity (Liu et al., 2017; Tsai et al., 2017; Feng et al., 2022) and can protect mesenchymal stem cells from OH-induced oxidative stress (Liu et al., 2016). It should be mentioned that mesenchymal stem cells can repair damaged cells, including damaged gastrointestinal mucosal tissue (Xiang et al., 2022). Thus, we speculate that SGN may have potential anti-GU ability. To date, gastroprotective effects of SGN on ethanol-induced GU and its underlying mechanisms have also not been evaluated. More importantly, the mechanism of action of SGN on GU should be elucidated to promote the clinical application of SGN.

TCM is a multi-component, multi-target, and multi-pathway treatment that achieves its particular protective pharmacological activity by modulating the biological network of body systems (Zhao et al., 2010). Therefore, it is difficult to detect the precise mechanisms of TCM via the conventional experimental method alone. Consequently, new and appropriate approaches are needed to systematically and comprehensively assess the mechanisms of Chinese materia medica. Metabolomics and network pharmacology can efficiently and systemically determine the molecular and pharmacological mechanisms due to the rapid advancement of analytical techniques and bioinformatics (Wang et al., 2019; Zhang et al., 2020). Unlike the earlier reductionist “one drug, one target” method, metabolomics and network pharmacology are based on the fact that numerous active ingredients interact with multiple diverse genes or proteins, similar to TCM. Metabolomics and network pharmacology can reflect and illustrate the interactive relationship between multiple drugs, targets, and diseases. Meanwhile, metabolomics combining chemometrics and multivariate statistical analysis methods can comprehensively analyze metabolites *in vivo* and characterize differential biomarkers to explore the correlation between metabolites and the physiological and pathological changes in the organism under drug treatment (Lucarelli et al., 2015). Network pharmacology abstracts the relationship into a network model and illustrates the action of drugs on the human biological network from a systematic perspective (Yang et al., 2020). In the present study, a metabolomics and network pharmacology approach were performed to investigate the impact of SGN on AGU, induced with ethanol, to clarify its medical value. Additionally, a potential novel insight was provided to systematically investigate the mechanism of action of SGN.

## 2 Materials and Methods

### 2.1 Materials and Reagents

SGN was obtained from the Yanbao Ecological Agricultural Farmers Professional Cooperative of Yong’an City, Fujian Province (voucher specimen NO20190813) and was identified as the dry whole plant of *Sarcandra glabra* (Thunb.) Nakai (Chloranthaceae) by Professor Qianfeng Gong of the Jiangxi University of Chinese Medicine. Chlorogenic acid reference substance (catalog number 110753-201716, purity ≥99.3%), rosmarinic acid reference substance (catalog number 111871-201505, purity ≥98%), and isofraxidin reference substance (catalog number 110837-201608, purity ≥98%) were obtained from the National Institutes for Food and Drug Control (Beijing, China). Neochlorogenic acid (catalog number BCTG-0231, purity ≥98%), cryptochlorogenic acid (catalog number BCTG-0210, purity ≥98%), caffeic acid (catalog number BCTG-0286), purity ≥98%), and astilbin (catalog number BCTG-0233, purity ≥98%) were acquired from the China National Engineering Research Center for Solid Preparation Manufacturing Technology (Jiangxi, China). Rat Tumor Necrosis Factor (TNF-α) ELISA Kit (catalog number: CSB-E11987r), Rat Interleukin 6 (IL-6) ELISA Kit (catalog number: CSB-E04640r), and Rat Prostaglandin E2 (PGE2) ELISA Kit (catalog number: CSB-E07967r) were provided by CUSABIO BIOTECH CO. Ltd. The myeloperoxidase (MPO) assay kit (catalog number: A044-1-1), superoxide Dismutase (SOD) assay kit (catalog number: A001-3-2), catalase (CAT) assay kit (catalog number: A007-1-1), malondialdehyde (MDA) assay kit (catalog number: A003-1-2), and nitric oxide (NO) assay kit (catalog number: A013-2-1) were purchased from Nanjing Jiancheng Bioengineering Institute. Methanol (CAS number: 67-56-1), acetonitrile (CAS number:75-05-8), ammonium acetate (CAS number: 631-61-8), and ammonium hydroxide (CAS number: 1336-21-6) were LC-MS grade. All other reagents and chemicals were of analytical grade.

### 2.2 Sample Preparation

#### 2.2.1 Preparation of Serum Samples of Rats

The serum sample (100 μL) was transferred to an EP tube; then, 400 μL of extract solution (acetonitrile: methanol = 1:1, containing isotopically labeled internal standard mixture) was added. The samples were vortexed for 30 s, sonicated in an ice-water bath for 10 min, and incubated at −40°C for 1 h to precipitate proteins. The samples were then centrifuged at 12,000 rpm and 4°C for 15 min to obtain the supernatant. The supernatant was transferred to a fresh glass vial for analysis. The quality control (QC) sample was prepared by mixing an equal aliquot of the supernatants from all the samples.

#### 2.2.2 Preparation of SGN Extract (Liu and Chen, 2016)

Eight times the volume of water was added to SGN herbal pieces (400 g) to soak for 1 h; they were decocted for 1 h, and six and four times the volume of water were added prior to them being decocted for 0.5 h. The solution was filtered with absorbent cotton after cooling. The filtrates were combined and evaporated to obtain a crude drug concentration of 1 gmL^−1^.

#### 2.2.3 High-Performance Liquid Chromatography for the Determination of Main Components

HPLC was performed using a Waters 2695 HPLC with quaternary solvent manager, column thermostat, diode array (PDA) detector, and Empower 3 chromatographic workstation (Waters Company, United States). A chromatographic Diamonsil C_18_ column (250 × 4.6 mm, 5 μm) with a mobile phase consisting of acetonitrile and 0.1% formic acid aqueous solution was used (flow rate, 0.8 ml/min; column temperature, 35°C, and elution as follows: 0–5 min, 6%–10% acetonitrile; 5–32 min, 10%–12% acetonitrile; 32–45 min, 12%–20% acetonitrile; 45–78 min, 20%–35% acetonitrile; 78–80 min, 35%–6% acetonitrile). The detection wavelength was 330 nm. The standard compounds were used for standard quantitative analysis.

### 2.3 Establishment of an Ethanol-Induced AGU Model

Male Sprague-Dawley (weight: 180–200 g) rats were obtained from Hunan SJA Laboratory Animal Co., Ltd. (Changsha, China) (License NO. SYXK 2019-0004). The animals were kept in an isolated room at 22°C ± 2°C and 55% ± 10% relative humidity with a 12-h light-dark cycle following the Jiangxi University of Chinese Medicine guidelines. The rats were randomly separated into three groups: SGN group (*n* = 8), model group (M) (*n* = 8), and normal control (NC) group (*n* = 6). Rats in the SGN group were orally administered SGN decoction (10 gkg^−1^), and those in the NC and M groups were given distilled water (10 ml kg^−1^ d^−1^) for seven consecutive days.

Rats were fasted but permitted access to water for 24 h before modeling. Rats in the NC group were given distilled water via gavage (5 ml kg^−1^), and the M group and SGN group were given an equal volume of anhydrous ethanol 1 h after the last administration for modeling. After 1 h, rats in each group were anesthetized and their blood was collected via the abdominal aorta. Their stomachs were photographed and removed immediately (Yoo et al., 2020; Yeo et al., 2021). This experiment was approved by the Experimental Animal Ethics Committee of Jiangxi University of Traditional Chinese Medicine (Animal Ethics Committee No. JZLLSC 2020_0108) and performed following the guidelines of the Chinese Ethics Committee.

### 2.4 Analysis of Pathology and ELISA of Rat Stomachs

The collected rat stomach tissues were cut symmetrically. Half of the tissues were put in a 4% paraformaldehyde solution for fixation for 24 h, then washed with flowing water to remove the fixative. The tissues were transferred to a 70% ethanol solution for storage. The tissues were successively put in 70%, 85%, 90%, and 100% ethanol for gradient dehydration until the tissue blocks were completely dehydrated and transparent. The tissues were then embedded, sectioned (6 μm), and HE-stained. A 100 × microscope was used to obtain the cytopathological image of gastric mucosal tissue (Lívero et al., 2016). The other half of the tissue was washed with pre-cooled PBS and weighed, and the corresponding volume of PBS (1 g: 9 ml) was added. The tissues were then ground on ice, frozen, and thawed repeatedly. The tissues were centrifuged at 5,000 r/min for 10 min to obtain a gastric tissue homogenate. The supernatants were assessed following the instruction of ELISA kits.

### 2.5 Metabolomic Analysis of Rat Serum

#### 2.5.1 UHPLC-QE-MS Analysis of the Rat Plasma

UHPLC system (Vanquish, Thermo Fisher Scientific) with a UPLC BEH Amide column (2.1 × 100 mm, 1.7 μm) coupled to a Q Exactive HFX mass spectrometer (Orbitrap MS, Thermo) was used for LC-MS/MS analyses. The mobile phase contained 25 mmol/L ammonium acetate and 25 mmol/L ammonia hydroxide in water (pH = 9.75) (A) and acetonitrile (B). The elution gradient was as follows: 0–0.5 min, 95% B; 0.5–7.0 min, 95%–65% B; 7.0–8.0 min, 65%–40% B; 8.0–9.0 min, 40% B; 9.0–9.1 min, 40%–95% B; and 9.1–12.0 min, 95% B. The column and auto-sampler temperatures were 30°C and 4°C, respectively, and the injection volume was 3 μL.

The QE HFX mass spectrometer can acquire MS/MS spectra on information-dependent acquisition (IDA) mode via the acquisition software (Xcalibur, Thermo), which continuously evaluates the full scan MS spectrum. The ESI source conditions were as follows: sheath gas flow rate; 50 Arb, Aux gas flow rate; 10 Arb, capillary temperature; 320°C, full MS resolution; 60,000, MS/MS resolution; 7,500, collision energy; 10/30/60 in NCE mode, spray Voltage; 3.5 kV (positive) or −3.2 kV (negative).

#### 2.5.2 Data Preprocessing and Biomarker Identification

ProteoWizard was used to convert the raw data to the mzXML format. The data were processed with an in-house program developed using R and XCMS for peak detection, extraction, alignment, and integration. After obtaining the sorted data, several multivariate pattern recognition analyses were conducted. A principal component analysis (PCA) was used to find the projection method that best represents the original data through dimensionality and noise reduction, visually displaying the differences between samples in a multi-dimensional space. Orthogonal partial least squares method-discriminant analysis (orthogonal projections to latent structures-discriminant analysis, OPLS-DA) was used to further analyze the results for better visualization and subsequent analysis. OPLS-DA analysis was used to filter metabolites unrelated to the categorical variables and separately analyze the non-orthogonal and orthogonal variables to obtain more reliable metabolite differences. SIMCA software (V14.1, Sartorius Stedim Data Analytics AB, Umea, Sweden) was used for logarithmic (LOG) conversion and UV formatting processing on the data to obtain relevant group information. First, OPLS-DA modeling was conducted on the first principal component. The quality of the model was determined via the seven-fold validation (7-foldcrossvalidation) test. The R2X (the interpretability of the model to the categorical variable × ) and Q2 (the predictability of the model) were obtained after cross-validation and used to evaluate the validity of the model. The permutation test was conducted to randomly change the arrangement of the categorical variable Y many times to obtain different random Q2 and R2 values and further test the validity of the model. The variable importance in the projection (VIP) value was obtained using the OPLS-DA mode discriminant analysis, and the *p*-value was obtained via the Student’s *t*-test analysis of standardized peak area. The different metabolites in each group were screened at *p* < 0.05 and VIP >1. MS1 mass spectrometry data with accurate molecular masses were recognized by searching Metlin, HMDB, and KEGG pathway databases, while identification of secondary spectra was based on the XCMS program package and the laboratory’s self-built database. The significantly different metabolites were identified. Finally, the metabolic pathways of the identified metabolites were obtained from MetaboAnalyst 5.0 (https://www.metaboanalyst.ca/) (Pang et al., 2021).

### 2.6 Network Pharmacology Analysis

#### 2.6.1 The Specific Operation of Network Pharmacology Analysis

Network pharmacology was used to comprehensively and systematically determine the material basis for the treatment of AGU using SGN, the relationship between metabolites and target proteins, and the mechanism of SGN treatment. The main steps were as follows: 1) The source of the active ingredients in SGN: 1) Traditional Chinese Medicine Systems Pharmacology Database and Analysis Platform (TCMSP) was used to obtain active ingredients. Oral bioavailability (OB) >30% and drug similarity (DL) >0.18 were used as the screening criteria. 2) The other chemical components in SGN were obtained via HPLC. 3) Prediction of component targets: The canonical SMILES structural formula of the active ingredients of SGN was obtained from the PubChem (https://pubchem.ncbi.nlm.nih.gov/) database. Swiss Target Prediction (www.swisstargetprediction.ch/) and DrugBank (https://go.drugbank.com/) were used to predict the targets of the active ingredients. 4) Disease target prediction: The targets of GU were obtained from the Genecards (https://www.genecards.org/) and DisGeNET (http://www.disgenet.org/) databases. 5) Establishment of Protein-Protein Interaction (PPI) network: The intersection targets of components and disease were imported to STRING 10.5 (https://string -db.org) to obtain the protein interaction network (PIN). 6) ClueGO (plug-in in Cytoscape software) was used to visually display the Gene Ontology (GO) enrichment and KEGG pathway on potential targets. The key signal pathways for SGN treatment of GU were screened out. This software can be used as a mutual verification of metabolic pathways obtained via metabolomics or complements of each other. 7) Component-reaction-enzyme-gene network was obtained by importing the metabolites obtained from metabolomics analysis into MetScape 3.1.3 [MetScape (http://metscape.ncibi.org/)]. Metscape is a visual Cytoscape software plug-in that can detect the relationship among metabolites, proteins, and corresponding regulatory genes in the human metabolic network. It can also interpret metabolome data. 8) The key metabolites and proteins were identified by integrating the network obtained in step 6 with hub genes and metabolic pathways.

### 2.7 Integrated Metabolomics and Network Pharmacology Analysis

To comprehensively understand the mechanism of SGN against AGU, the differential metabolites identified by metabolomics were imported into MetScape, and pathway-based analysis was performed to obtain the Compound-Reaction-Enzyme-Gene network. The targets predicted by MetScape were matched with the targets predicted by network pharmacology, and the hub genes were acquired.

#### 2.7.1 Molecular Docking Verification of Key Targets

In order to better illuminate the binding activity between the potential targets of SGN to prevent AGU with the corresponding active ingredients, the critical active ingredients were obtained from the analysis of the “ingredient-target” network, while the key targets were acquired through the joint strategy, and the binding ability of them were tested for molecular docking verification. The crystal structures of the core target protein receptor were downloaded from the RCSB PDB database (https://www.rcsb.org/) and PyMoL software was applied to delete the target irrelevant protein receptor ligands and non-protein molecules (such as water molecules). AutoDock Tools (version 1.5.6) was used to perform routine pretreatment of the target protein receptors and small ligand molecules, the Grid Box with the ligand was used as the center, and the docking active site and binding energy were gained using the Autogrid module and by performing molecular docking, respectively. Generally, lower energy means that the conformation of the compound molecule and the receptor may be more stable and the docking result may be more reliable. The component with the lowest docking binding energy to the target protein was selected to visualize the results by PyMoL.

#### 2.7.2 Western Blot to Validate the Results of the Integrated Analysis

Specific information on grouping and handling of rats can be found in the Supplementary file. RIPA lysis buffer was used to extract protein from rat stomach tissue, and the protein concentration was determined and calculated using the bicinchoninic acid (BCA) method. A Bio-Rad electroporation instrument transfer membrane was used to separate the proteins through SDS-PAGE using the same amount of protein per sample (30 μg/sample). The membranes were blocked for 2 hours at room temperature, treated with 1:500 diluted primary antibody, and kept overnight at 4°C. The membrane was washed three times with TBS-T for 10 min each time, then treated with a 1:5,000 dilution of horseradish peroxidase-labeled secondary antibody, and incubated at room temperature for 2 hours; the membrane was washed three times with TBS-T for 10 min each time. ECL luminescent agent was used for 1 minute in a dark room before being exposed. A gel imaging analysis system was used for quantitative analysis, GAPDH was used as an internal reference, and the ratio of the gray value of the target protein to the gray value of GAPDH was used to reflect the expression of related proteins.

### 2.8 Statistical Analysis

SPSS version 21.0 (SPSS Inc. Chicago, Illinois) was used for all statistical analyses. The data are expressed as mean ± SD. One-way ANOVA was used for the significance test. The SNK test was used for multiple comparisons when the variance was uniform, while the Tamhane test was used if the variance was uneven. A *p*-value < 0.05 was considered a statistically significant difference.

## 3 Results

### 3.1 HPLC for the Determination of Main Components in SGN

The chromatogram of SGN decoction is shown in Supplementary Figure S1. The liquid chromatogram shows that there are mainly 7 chromatographic peaks. A total of 7 compounds have been identified by comparing these peaks with the chemical reference, namely neochlorogenic, chlorogenic, cryptochlorogenic, caffeic, and rosmarinic acid, as well as isofraxidin and astilbin.

### 3.2 Effects of SGN on the Histopathological Morphology of Rat Stomachs

**FIGURE 1:**
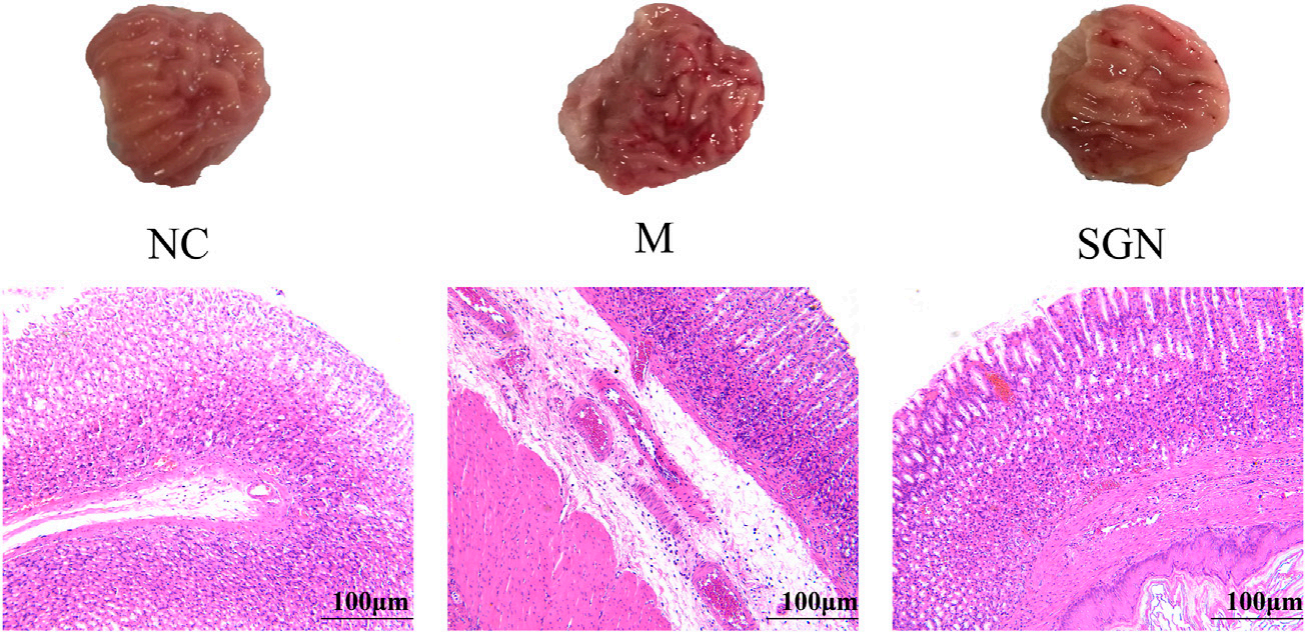
Pathological section of rat stomach tissue in each group ( × 100). Black arrow: edema in the submucosal layer, eosinophil infiltration, damage to the glandular mucosal structure. Green arrow: bleeding on the mucosal surface, vasodilation, and congestion in the muscle layer.

The rats in the M group showed edema in the submucosal layer, bleeding on the mucosal surface, severe damage to the glandular mucosal structure, vasodilation, and significant congestion in the muscle layer compared with those in the normal group. Slight lamellar bleeding was also seen in the SGN group. However, congestion in the gastric mucosa, bleeding, and mucosal damage was significantly reduced in the SGN group compared with those in the M group. SGN decoction reduced GU bleeding and protected the gastric mucosa. The pathological sections results showed that the epithelial cells of the model group were defective and had several inflammatory cell infiltrations compared with those in the NC group. The gastric mucosal layer of rats was completely repaired, and the inflammatory exudate and inflammatory cells were significantly reduced in the SGN group compared to those in the M group (See Figure 1).

### 3.3 Analysis of Related Cytokine Levels by ELISA Kits

**FIGURE 2:**
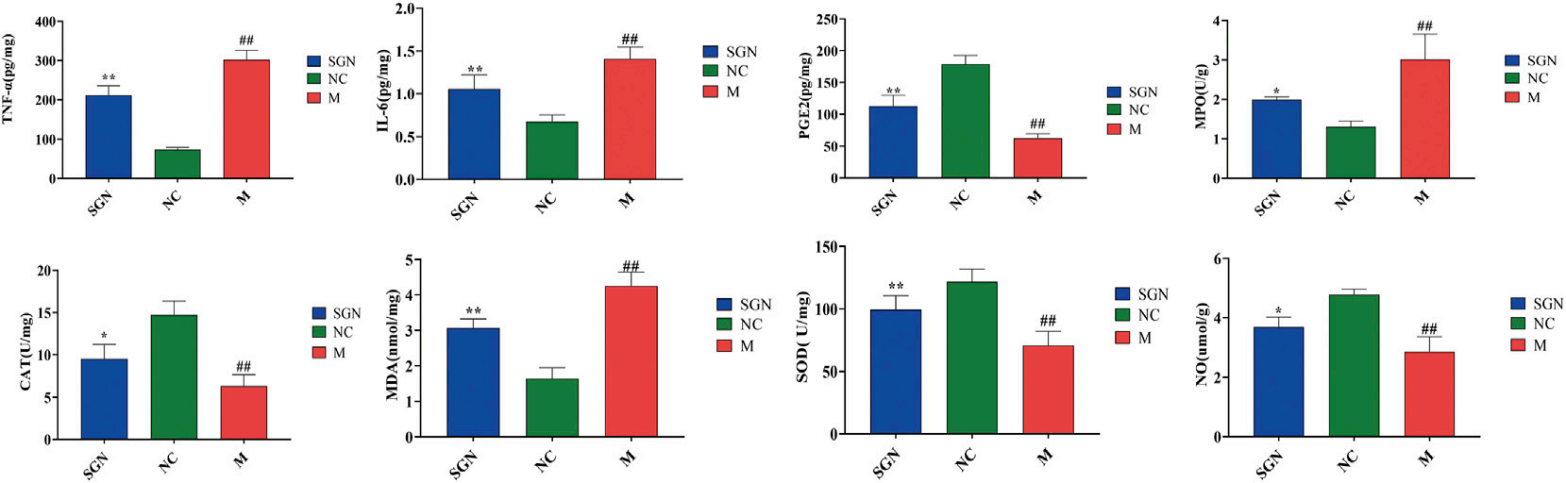
The effect of SGN on TNF-α, IL-6, PGE-2, NO, MPO, MDA, CAT, and SOD in gastric homogenate (mean ± SD, *n* = 6). Note: Compared with NC group, ##*p* < 0.01, compared with model group, *p < 0.05, **p < 0.01.

The effect of SGN on inflammatory and oxidative stress indexes in rats with ethanol-induced GU is shown in Figure 2. The ethanol gavage significantly increased the levels of inflammatory factors TNF-α and IL-6 in the gastric tissue of the rats (*p* < 0.01), while the contents of PGE-2 and NO were significantly decreased compared with those in the NC group (*p* < 0.01). Furthermore, the oxidative stress index, MDA content, and MPO enzyme activity were significantly increased (*p* < 0.01), while the enzyme activity of CAT and SOD was significantly decreased (*p* < 0.01). SGN gavage significantly increased the content of PGE2 and NO in rat gastric tissue compared with that in the M group (*p* < 0.05). SGN gavage also reversed the downward trend of TNF-α and IL-6 (*p* < 0.01). SGN decoction improved the oxidative stress level of the gastric tissue of the rats. The SOD enzyme activity and CAT content increased, while the MPO enzyme activity was significantly inhibited and the MDA content level was significantly decreased (*p* < 0.05).

### 3.4 Integrated Strategy Based on Metabolomics and Network Pharmacology

#### 3.4.1 PCA of Rat Serum in all Groups

**FIGURE 3:**
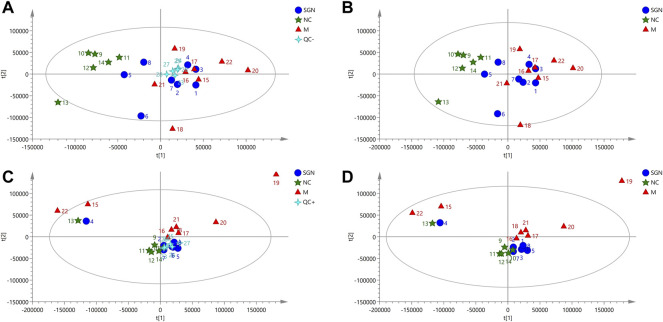
PCA score plots of serum in positive and negative modes.

The PCA results of SGN extract on serum metabolites of ethanol-induced AGU model rats showed that all QC samples were similar and that they were well gathered near the origin. These results indicate that the detection platform was stable, the instrument precision was good, and the method was reliable (Figures 3A,C). The distance was furthest between the M and NC group, indicating that the serum metabolites of the M group was significantly changed after the model development. The SGN group was between the M group and NC groups, indicating that SGN reversed the cause after administration. The disturbance and deviation of serum metabolites caused by the model are shown in Figures 3B,D. The total ion current diagram is shown in Supplementary Figure S2.

#### 3.4.2 OPLS-DA Analysis of Rat Serum in Each Group

**FIGURE 4:**
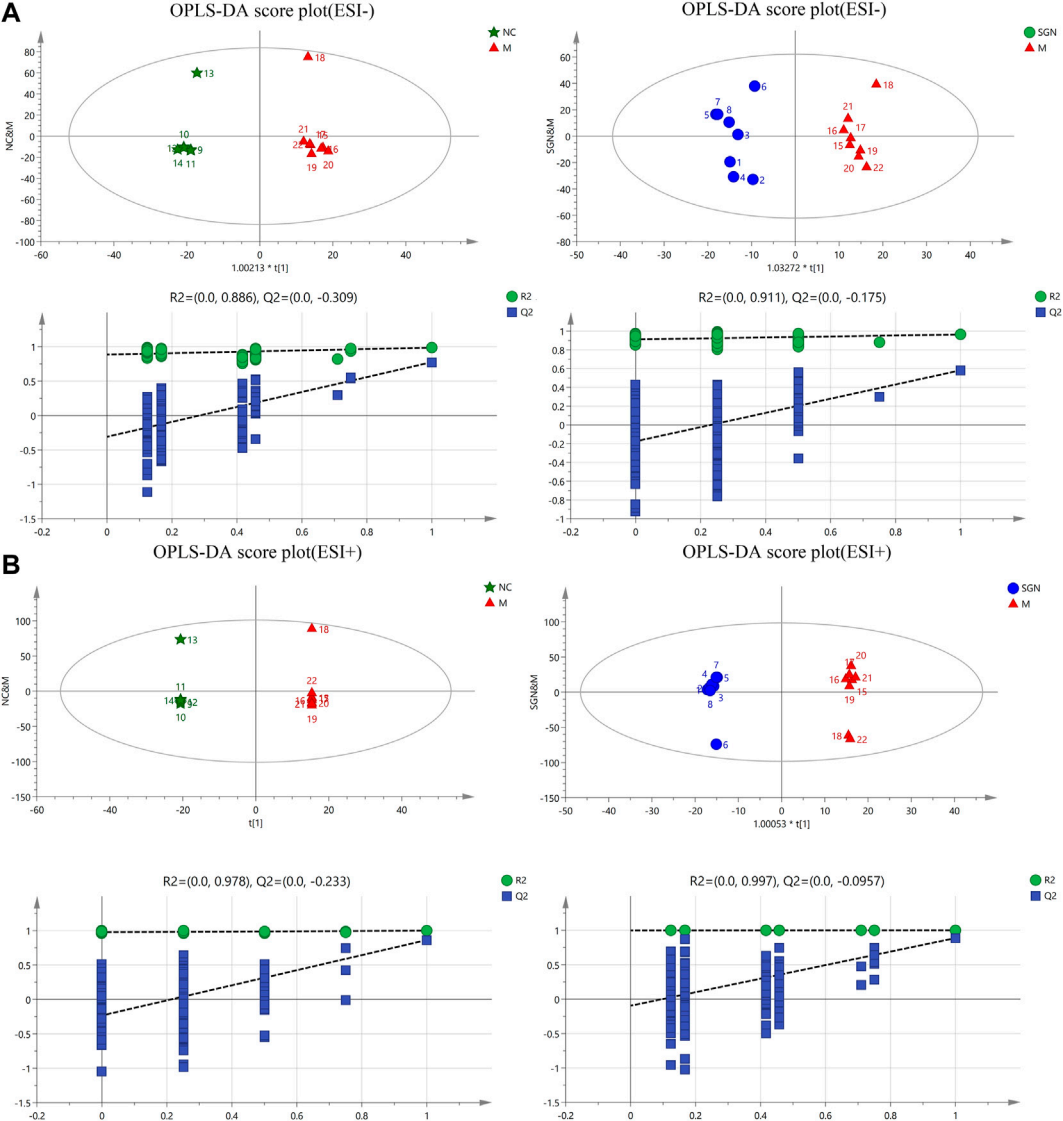
OPLS-DA score plots and corresponding model validation plot of rat serum samples in positive and negative modes.

The OPLS intrinsic structure projection method was used to analyze each experimental group to eliminate the influence of irrelevant noise, such as intra-group differences and cross factors. OPLS-DA analysis was used to filter and classify metabolites. Orthogonal variables with uncorrelated variables and separate analyses of non-orthogonal and orthogonal variables were conducted to obtain a more reliable correlation between metabolite differences and experimental groups. The score of OPLS-DA indicated that all samples were within the 95% confidence interval (Hotelling T2 ellipse). The cumulative values of R2Y and Q2 were greater than 0.6, indicating that the OPLS-DA model was suitable for determining the difference between the two groups of samples. The difference further confirmed the significant changes in the relevant metabolic components of the ethanol-induced GU model rats (Figures 4A–1, 2 and B-1, 2). Finally, a 200-permutation test was used to discriminate the OPLS-DA model to verify its stability and reliability. The cross-validation (Q2) analysis revealed that the change in all data (R2) determined that the model did not overfit (Figures 4A–3, 4 and B-3, 4).

#### 3.4.3 Identification of Characteristic Metabolites in Rat Serum and Related Metabolic Pathway Analysis

**FIGURE 5:**
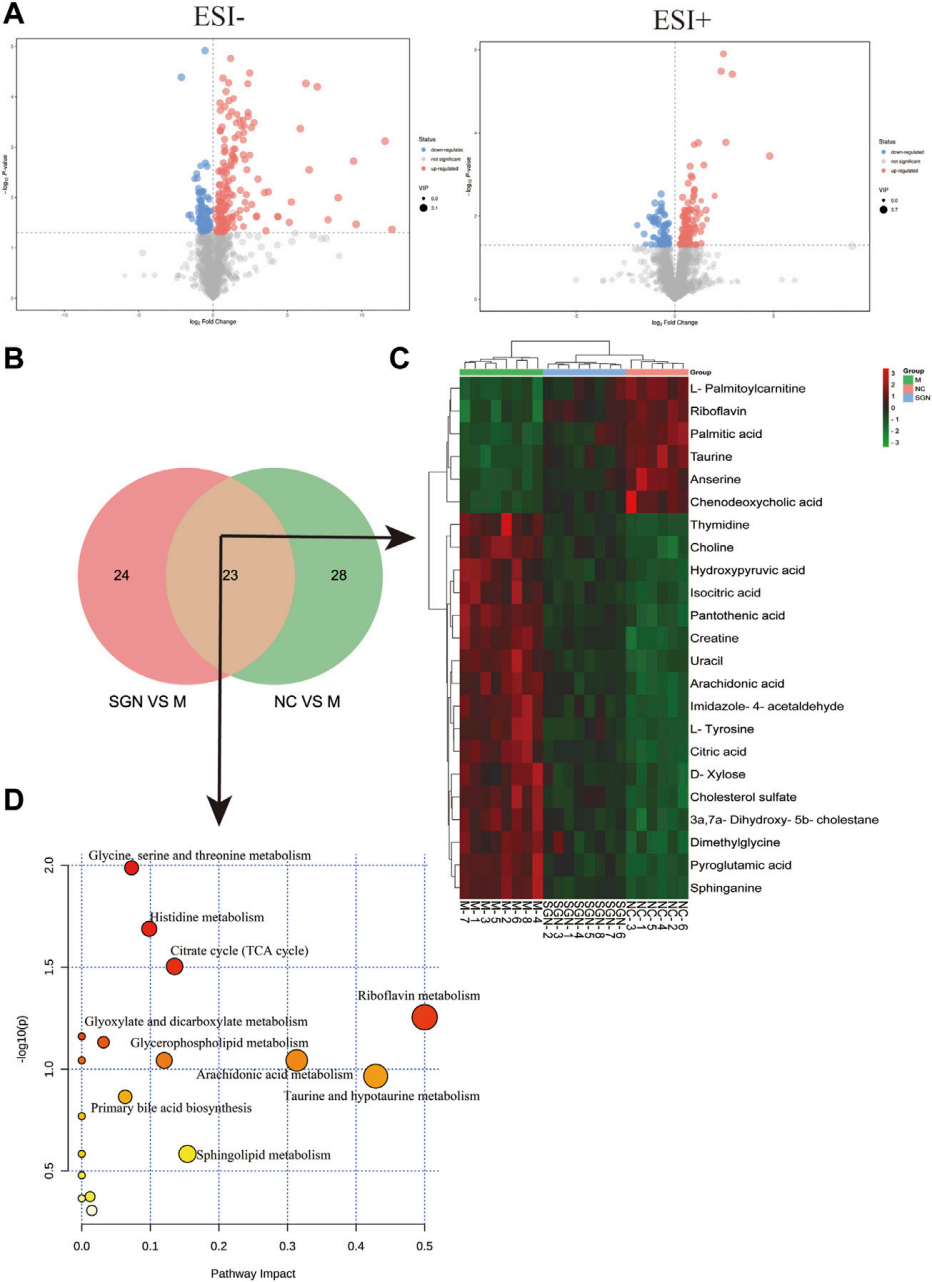
**(A)** Differential metabolite volcano plot of SGN VS. M in negative and positive ion mode. **(B)** Venn plot of common metabolites in the SGN and NC groups. **(C)** Heatmap of different endogenous metabolites. Rows represent the metabolites, and columns represent the corresponding group. **(D)** Serum metabolism pathway in ethanol-induced GU rats treated with SGN.

The metabolites with VIP score >1 and *p* < 0.05 were filtered and collected. The precursor ions and MS/MS fragments obtained via high-resolution UHPLC-QE-MS, and the metabolite information obtained from the HMDB, Metlin, and KEGG databases were used to determine if the error between the extracted mass value and the experimental mass value was less than 10 ppm. The metabolites that met the above conditions were identified as candidate biomarkers. Volcano-plots were used to further imply the changed metabolites (*p* < 0.05) among the different groups (Figure 5A). Finally, 23 metabolites (Table 1) were identified as candidate biomarkers (Figure 5B). The expression levels of the candidate biomarkers were assessed and compared to determine the expression of metabolites in the NC, M, and SGN groups (metabolite thermogram, Figure 5C). The color change on the heat map indicates the overall change in metabolites among the groups. For instance, the levels of arachidonic acid, sphingosine, and citric acid were higher in the M group than in the NC group. The abundance of arachidonic acid and sphingosine was lower in the SGN group than in the M group. The results indicated that SGN treatment had a reversal effect on most of the metabolites and were regulated to recover to levels similar to the NC group. The VIP value is indicated by the size of the circle, red indicates significant upregulation, and blue represents significant downregulation. Moreover, the related metabolic pathways were established by MetaboAnalyst 5.0 (Figure 5D).

**TABLE 1:** Differential metabolites identification of serum.

NO	Name	HMDB	VIP	*p*-value	Formula	m/z	FC	Retention time(s)	Adduct
1	Pantothenic acid	HMDB0000210	1.819954	0.004677	C_9_H_17_NO_5_	218.1027	1.556969	287.41	[M–H]^−^
2	Citric acid	HMDB0000094	1.777119	0.016476	C_6_H_8_O_7_	191.0191	2.478995	132.1855	[M–H]^−^
3	Isocitric acid	HMDB0000193	1.928636	0.001459	C_6_H_8_O_7_	191.0192	2.428676	101.09	[M–H]^−^
4	L-Tyrosine	HMDB0000158	1.439888	0.032418	C_9_H_11_NO_3_	180.0657	1.759687	322.721	[M–H]^−^
5	Dimethylglycine	HMDB0000092	2.052247	2.86E-06	C_4_H_9_NO_2_	102.0551	4.051005	343.131	[M–H]^−^
6	Taurine	HMDB0000251	1.919971	0.003001	C_2_H_7_NO_3_S	124.0064	1.460747	313.703	[M–H]^−^
7	Thymidine	HMDB0000273	1.343658	0.038894	C_10_H_14_N_2_O_5_	241.0824	1.523908	91.8446	[M–H]^−^
8	D-Xylose	HMDB0000098	1.840493	0.00347	C_5_H_10_O_5_	149.0446	1.507816	166.024	[M–H]^−^
9	Uracil	HMDB0000300	1.454253	0.004192	C_4_H_4_N_2_O_2_	111.019	1.416469	79.7947	[M–H]^−^
10	Pyroglutamic acid	HMDB0000267	1.484675	0.014249	C_5_H_7_NO_3_	128.0343	1.366563	317.204	[M–H]^−^
11	Arachidonic acid	HMDB0001043	1.394256	0.00403	C_20_H_32_O_2_	303.2322	1.307376	38.1969	[M–H]^−^
12	Cholesterol sulfate	HMDB0000653	1.39984	0.029981	C_27_H_46_O_4_S	465.3045	1.358335	26.0519	[M–H]^−^
13	Hydroxypyruvic acid	HMDB0001352	1.947684	0.003819	C_3_H_4_O_4_	103.0027	2.621461	97.4662	[M–H]^−^
14	Chenodeoxycholic acid	HMDB0000518	1.954149	0.013724	C_24_H_40_O_4_	391.2847	0.341368	169.658	[M–H]^−^
15	Anserine	HMDB0000194	1.964544	0.032861	C_10_H_16_N_4_O_3_	239.1145	1.892027	432.811	[M–H]^−^
16	Palmitic acid	HMDB0000220	1.701405	0.022209	C_16_H_32_O_2_	255.2323	1.422017	220.4415	[M–H]^−^
17	Creatine	HMDB0000064	1.828211	0.020049	C_4_H_9_N_3_O_2_	130.0612	1.449975	367.008	[M–H]^−^
18	L-Palmitoylcarnitine	HMDB0000222	1.740218	0.045599	C_23_H_46_NO_4_	401.3404	0.761472	201.0715	[M + H]^+^
19	3a,7a-Dihydroxy-5b-cholestane	HMDB0006893	1.93586	0.013094	C_27_H_48_O_2_	405.3709	1.601535	177.359	[M + H]^+^
20	Riboflavin	HMDB0000244	1.325923	0.048874	C_17_H_20_N_4_O_6_	377.144	1.435412	234.177	[M + H]^+^
21	Imidazole-4-acetaldehyde	HMDB0003905	3.003304	0.008703	C_5_H_6_N_2_O	111.0553	2.4061	79.35665	[M + H]^+^
22	Sphinganine	HMDB0000269	1.762494	0.049348	C_18_H_39_NO_2_	302.3038	1.488641	130.652	[M + H]^+^
23	Choline	HMDB0000097	2.379748	0.033347	C_5_H_14_NO	103.0993	1.774886	404.484	[M + H]^+^

### 3.5 Analysis of the Anti-GU Mechanisms of SGN Based on Network Pharmacology

#### 3.5.1 Network Analysis of Potential Active Ingredients and Candidate Targets of SGN

Active SGN ingredients (17) (Supplementary Table S1) were obtained following the procedure outlined in section 2.8. However, one had no target and was removed. A total of 245 potential targets were identified after removing the duplicate values. The targets were used to construct a candidate compound-candidate target network regulatory network in SGN (Supplementary Figure S4). The network had 262 nodes and 477 edges. The network graph also showed complex correlations between different components and targets. Analyze Network was used for network analysis to identify more relevant active ingredients. Quercetin, isofraxidin, and rosmarinic acid had the highest degree of connection with the target protein (Supplementary Table S2).

#### 3.5.2 Screening Hub Targets of SGN Against GU Based on a PPI Network

**FIGURE 6:**
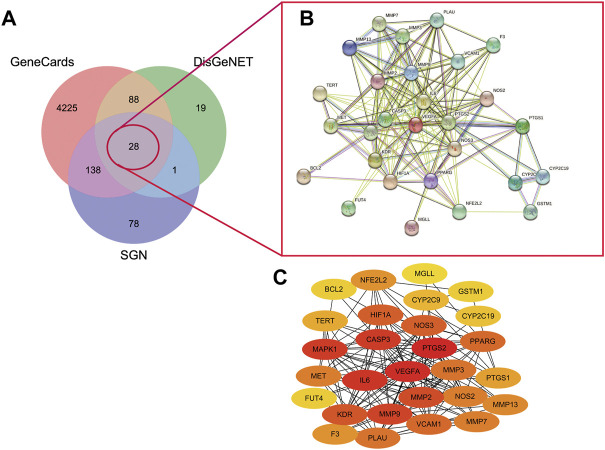
A. Venny diagram of targets of SGN with AGU.B. PPI network of SGN and GU overlap targets C. core targets plot. The redder the color mean more important of the targets.

A total of 136 and 4479 GU-related targets were obtained from DisGeNET and GeneCards databases, respectively. Out of these, 116 overlapped in the two databases. The 28 main targets were obtained by matching predicted targets of the components in the SGN with the GU-related targets (as shown in Figure 6A). The hub targets in the constructed PPI network (Figure 6B) were identified via cytoHubba (Figure 6C). The redder the color, the higher the degree value. PTGS2, VEGFA, CASP3, IL6, MMP2, MMP9, MAPK1, and KDR had a high degree value in the PPI network and play indispensable roles in network regulation (See Supplementary Table S5 for details).

#### 3.5.3 Enrichment Analysis of GO and KEGG Pathway for SGN Against GU

**FIGURE 7:**
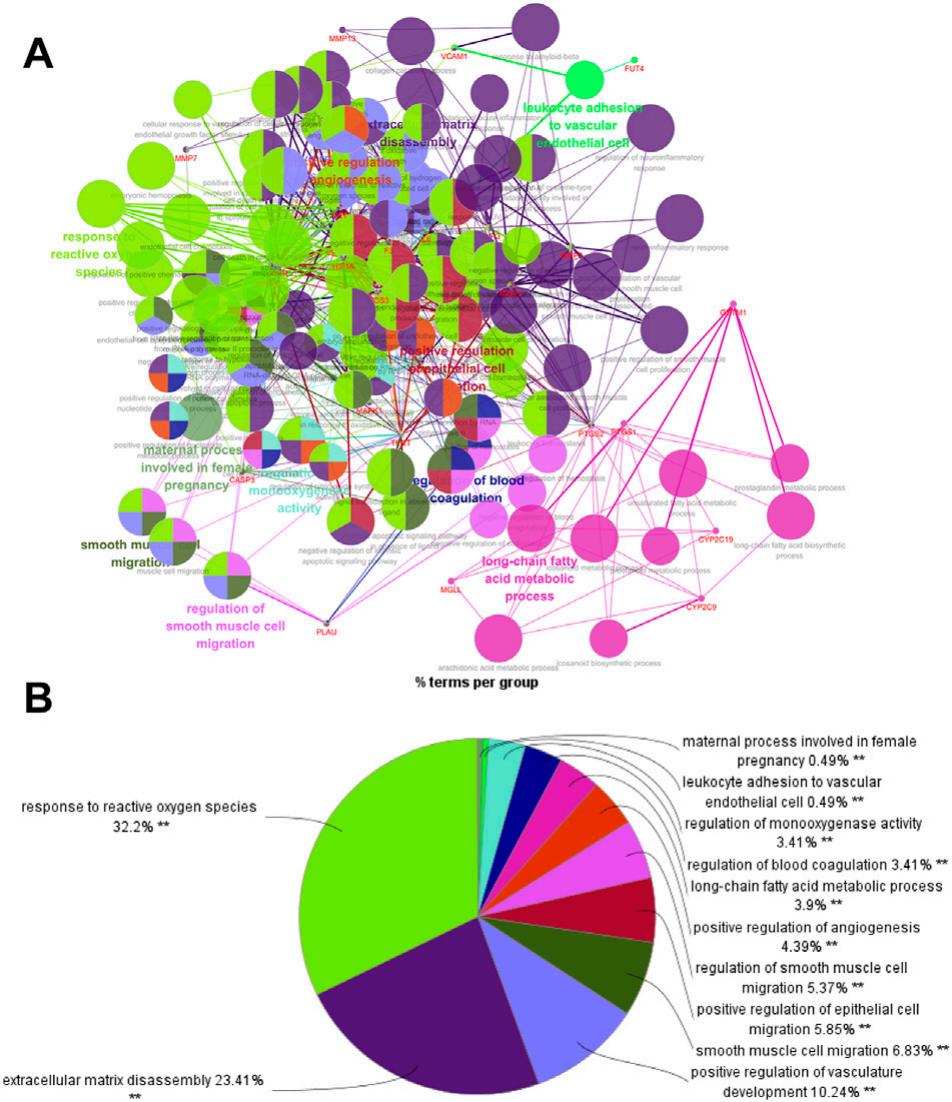
Biological process of GO enrichment analysis (from ClueGO).

**FIGURE 8:**
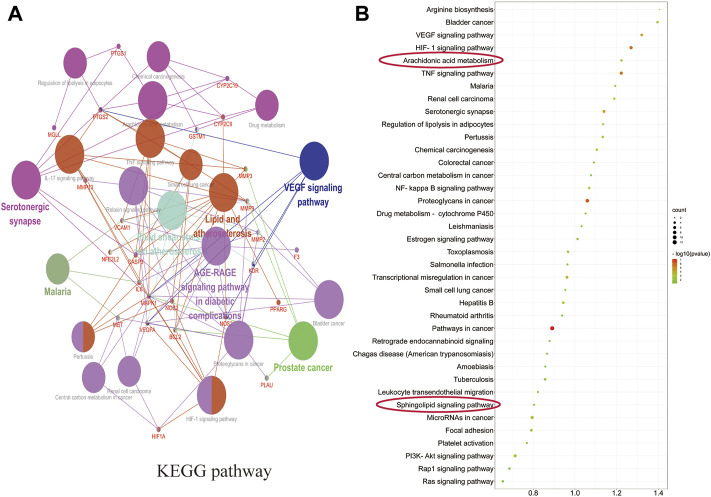
Pathways in KEGG enrichment analysis.

ClueGO was used for GO and KEGG function annotation and enrichment analysis on the 28 key target genes. The biological process network diagram of GO enrichment analysis is shown in Figure 7A. Each node represents an enrichment pathway, the line of the node indicates the number of genes shared among the pathways, and the color indicates the enrichment classification of the node. GO enrichment analysis showed that SGN is involved in 108 biological processes, including response to reactive oxygen species, leukocyte homeostasis, the release of cytochrome c from mitochondria, cell migration for sprouting angiogenesis, cytokine production for inflammatory response, regulation of acute inflammatory response, and prostanoid metabolic process. GO analysis also revealed that SGN is mainly involved in oxidative stress, immune system processes, and the regulation of defense responses in anti-GU (Figure 7B). Therefore, the etiology of ethanol-induced GU may be related to oxidative stress and immune response. KEGG functional annotation enrichment analysis suggested that SGN is involved in 38 pathways, including arachidonic acid metabolism, the HIF-1 signaling pathway, the TNF signaling pathway, the AGE-RAGE signaling pathway, and the sphingolipid signaling pathway (Figure 8). Figure 8A shows the enrichment analysis result of ClueGO, and Figure 8B shows the DAVID database result, as a supplement to the result of A. Therefore, SGN treats GU by regulating multiple biological processes and through the coordination of multiple pathways. Moreover, KEGG enrichment indicated that the regulatory mechanisms of the disease were interrelated and influenced each other. Enrichment analysis showed that malaria, pertussis, amoebiasis, hepatitis B, and cancers (prostate cancer, bladder cancer, and small cell lung cancer) were significantly enriched.

### 3.6 Integration Analysis of Metabolomics and Network Pharmacology

**FIGURE 9:**
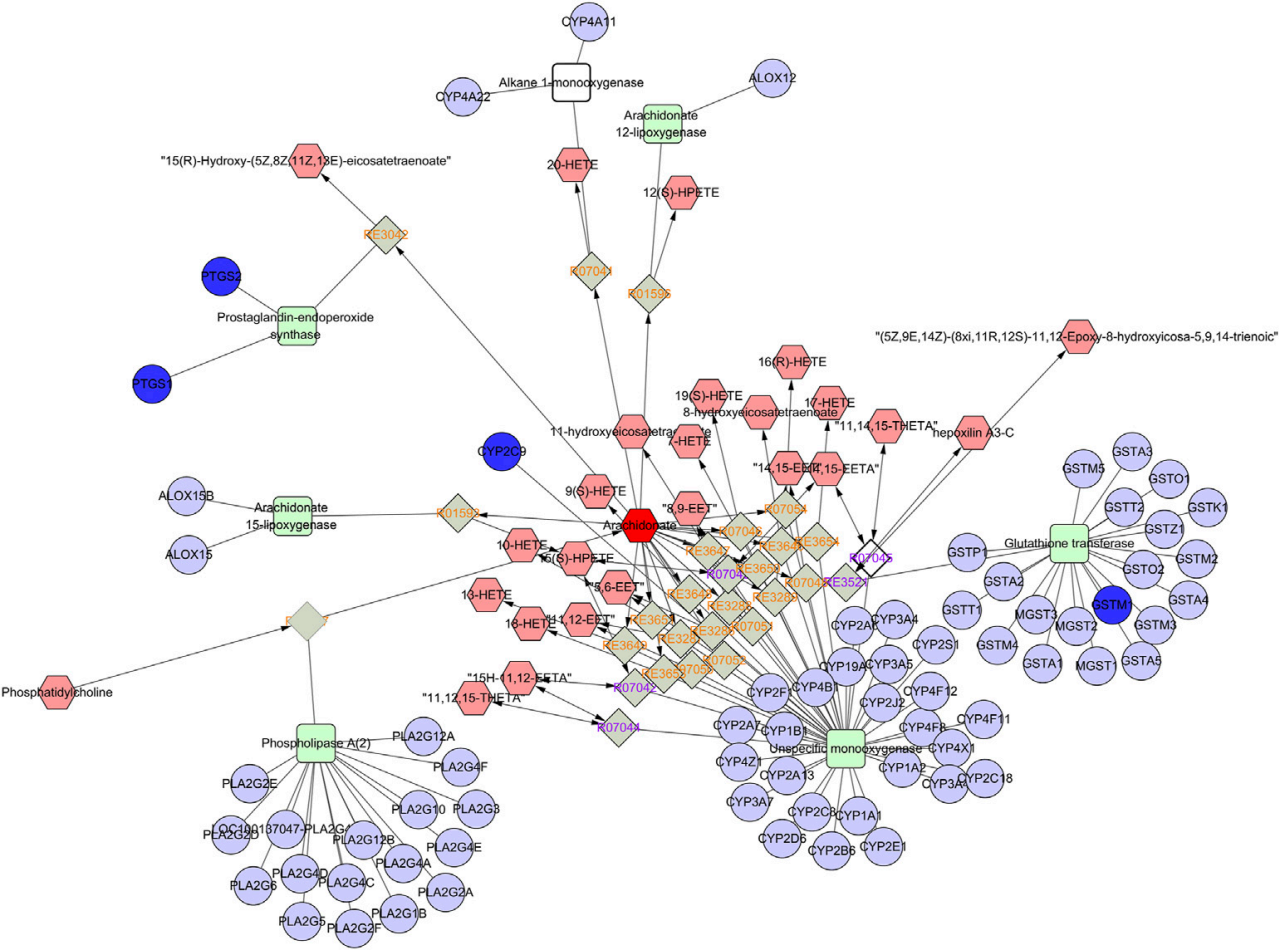
Part of compound-reaction-enzyme-gene network (Arachidonic acid metabolism). Red hexagons, gray diamonds, green rectangles, and purple circles represent active compounds, reactions, proteins and genes, respectively.

An interaction network was built by combining metabolomics with network pharmacology via MetScape (Figure 9) to fully understand the mechanism of SGN against GU. Eleven key targets, including PTGS2, MAPK1, KDR, and PTGS1, were identified. C00219 (arachidonic acid), C00114 (choline), and C00836 (sphinganine) were the major related key metabolites. Arachidonic acid, glycerophospholipid, and sphingolipid metabolism were the main pathways involved in the correlation analysis. These genes play a crucial role in the protective effect of SGN through the above pathways and the metabolites may be potential markers of GU; the overall compound-reaction-enzyme-gene network is shown in Supplementary Figure S5.

### 3.7 The Result of Molecular Docking

**FIGURE 10:**
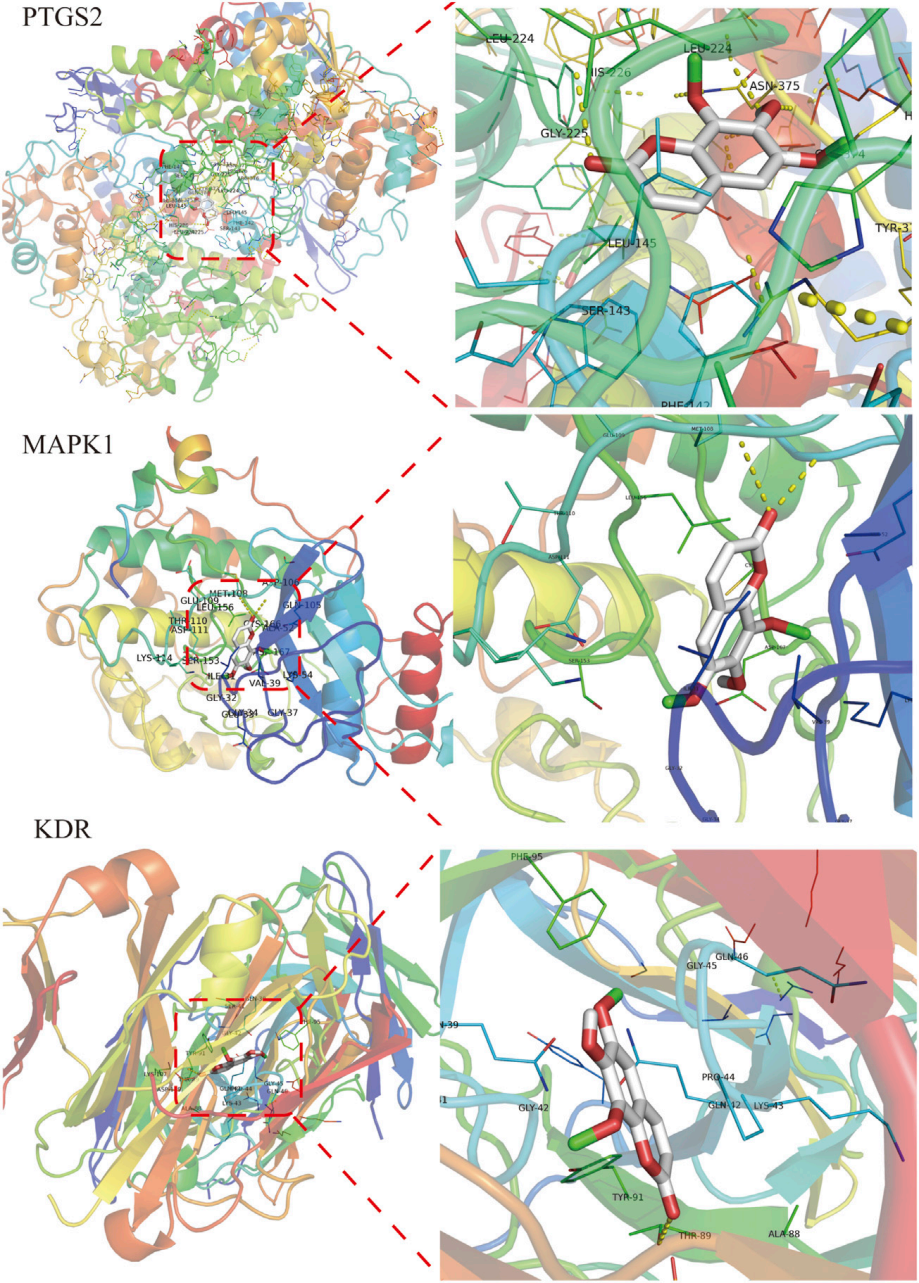
Docking results of isofraxidin with key targets.

Isofraxidin was finally selected for molecular docking based on the results of HPLC liquid phase component identification and the component-target network. The binding ability of isofraxidin was predicted with hub targets such as PTGS2, MAPK1, and KDR. The binding energy, which was less than −4.25 kcal mol^−1^, indicated that the ligand has a certain binding activity with the receptor; less than −5.0 kcal mol^−1^ implied good binding activity, while less than −7.0 kcal mol^−1^ suggested strong binding activity. The results showed that isofraxidin has good binding ability with PTGS2, MAPK1, and KDR, with binding energies of −6.0, −6.6, and −5.3 kcal mol^−1^, respectively, and its docking results were visualized, as shown in Figure 10.

### 3.8 Expression of Related Proteins Detected by Western Blotting

**FIGURE 11:**
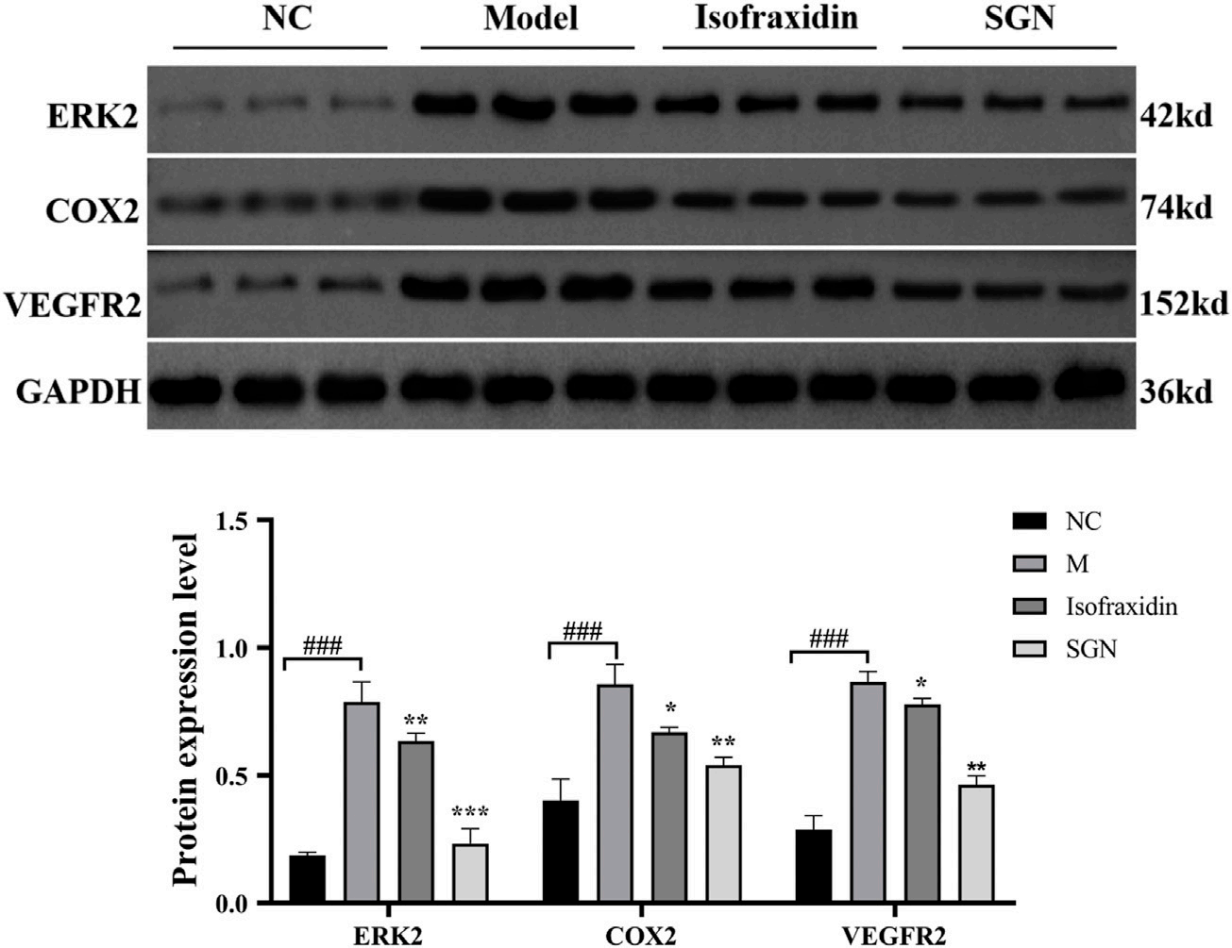
The expression of related proteins in each group of rats. The data presented are means ± SD (*n* = 3). ###*p* < 0.01 relative to the NC group. Compared with the M group, *p < 0.05, **p < 0.01 and ***p < 0.01.

Compared with the NC group, MAPK1, cyclooxygenase (COX-2), and VEGFR2 protein expressions were significantly increased in the M group (*p* < 0.001). While compared with the M group, the isofraxidin group could significantly reduce the protein expression of MAPK1, COX-2, and VEGFR2 (*p* < 0.01 or *p* < 0.05), and the SGN group could significantly reduce the expression levels of MAPK1, COX-2, and VEGFR2 (*p* < 0.001 or *p* < 0.01). See Figure 11.

## 4 Discussion

In the present study, an alcohol-induced AGU model was used to investigate the potential protective mechanism of SGN on GU. The pharmacodynamic evaluation was conducted using traditional pharmacodynamic indicators, such as histopathological examination and related cytokine levels. Meanwhile, conjoint analysis of metabolomics and network pharmacology was also used for further comprehensive analysis of the active ingredients of SGN acting on GU. UHPLC-QE-MS metabolomics technology was used to detect the collected serum samples of rats in each group. Multivariate statistics and other metrology were used for screening differentially expressed metabolites and to analyze related metabolic pathways. Metabonomics identified 23 serum biomarkers related to GU. Additionally, SGN treatment had a significant reverse effect on 8 rat serum pharmacodynamic markers. The protective effect of SGN on GU could be through the regulation of the serum metabolic profile. Network pharmacology showed that SGN acted on multiple targets, such as PTGS2, MAPK1, and KDR, through multiple active ingredients, including quercetin, isofraxidin, and rosmarinic acid. Furthermore, one target had multiple components. SGN was also associated with multiple signaling pathways related to cell inflammation and immunity, signal transduction, metabolism, apoptosis, and differentiation.

### 4.1 The Mechanism of Action of SGN Against AGU Based on Metabolomics

It has been reported that the occurrence of alcohol-induced GU is mainly related to oxidative stress, inflammation, and lipid peroxidation (Kobayashi et al., 2001; Zhang et al., 2019; Ahmed et al., 2020). Oxidative stress is the excessive production of free radicals when the body is subjected to various harmful stimuli (radiation, ischemia, hypoxia, etc.,) which the antioxidant system cannot effectively and efficently remove, causing an imbalance between the oxidation and antioxidant system, thus leading to tissue damage (Tan et al., 2018). Oxidative damage can lead to gastric mitochondrial dysfunction and directly induces ATP production. The TCA cycle is essential for carbohydrate, fat, and protein metabolism and crucial for energy metabolism to produce ATP. Succinic, citric, fumaric, and isocitric acid are the main products of the TCA cycle. Excessive alcohol consumption can inhibit the activity of mitochondria in the stomach, which leads to the production of reactive oxygen species (ROS) and an increased oxidative stress response, further mediated by the massive release of inflammatory mediators, causing submucosal microvascular circulation disorders and insufficient blood supply (Cappel et al., 2019). In addition, clinical studies have shown that histidine exhibits anti-inflammatory, antioxidant, and free radical scavenging activities.

Interestingly, SGN can also alleviate the body’s oxidative damage by regulating the metabolism of citric, isocitric, and hydroxypyruvic acid. Additionally, ethanol can infiltrate neutrophils in the gastric mucosa and release MPO, oxygen-free radicals, active oxidative metabolites such as superoxide anion (O_2_^−^), and protease. These can adhere to the vascular endothelium to cause large blood vessel occlusion, thus leading to mucosal damage (Zhao et al., 2016). Additionally, MPO-catalyzed reaction generates excessive oxidants (HOCl, 3-chlorinated tyrosine, tyrosyl, nitrotyrosine, etc.,) which can cause oxidative stress and oxidize when it exceeds the defense reaction of local antioxidants, thus leading to tissue damage (Goud et al., 2021). SOD scavenges free radicals when its level is regarded as an intuitive indicator of aging and death (Schatzman and Culotta, 2018). CAT is an antioxidant enzyme that can prevent the toxic effects of excessive hydrogen peroxide on cells (Rakotoarisoa et al., 2019). These indicators are commonly used to measure oxidative damage in the body. In the present study, SGN alleviated oxidative stress in rats with GU by promoting the antioxidant enzyme activity (SOD), increasing CAT content, and balancing excess oxidation products (MPO) in stomach tissues, similar to the results presented by most TCMs that protect the stomach (Byeon et al., 2018).

Notably, ROS promotes the development of GU (Perez et al., 2017), which may attack the polyunsaturated fatty acids in the phospholipids of biological membranes, further causing lipid peroxidation. The free radicals can damage the gastric mucosa, which can be indicated by the increased content of its metabolite MDA in the tissues (Busch and Binder, 2017). Additionally, we found that arachidonic acid (AA) and sphinganine levels were significantly elevated in the M group; however, SGN suppressed the elevation of the two. According to literature reports, AA is an omega-6 polyunsaturated fatty acid that is most widely distributed in the body and has important biological activities. When the inflammatory substance invades the organism, the phospholipase A2 (PLA2) in the body is activated to catalyze the phospholipid two-position acyl group. Hydrolysis breaks down AA into its free form and releases it into the cell fluid. Free AA is mainly metabolized by three pathways, COX, lipoxygenase (LOX), and cytochrome P450 (CYP450), to produce a series of different substances with strong biological activity (Liu et al., 2019). Sphingolipids are structural components of cell membranes, associated with a host of metabolic networks in the body, and play signaling roles that regulate a multitude of activities, including mitochondrial function and cell death (Manzanares-Estreder et al., 2017). This evidence suggested that ethanol treatment induces oxidative stress in rat gastric tissue, which further induces inflammation and metabolic disturbance and may eventually lead to apoptosis. Our results revealed that the treatment of SGN may play a preventive role in the development of GU.

Incidentally, the protective mechanism of SGN also involves related amino acid metabolism, and we speculate that these amino acid metabolites act by attenuating the destruction of antioxidant defense systems, glutathione (GSH) and NO production in tissues, and neutrophil infiltration to protect rat gastric tissue from oxidative damage. Previous studies have shown that the antioxidant effect of taurine restores the homeostasis of the redox microenvironment in rats by promoting the content of glutathione, accomplishing gastroprotection against indomethacin-induced gastric injury (Motawi et al., 2007). Flavin mononucleotide (FMN) and flavin adenine dinucleotide (FAD) are the active forms of riboflavin (vitamin B2) in the body (Ashoori and Saedisomeolia, 2014). Glutathione reductase (GR) is a FAD enzyme. The redox cycle of GSH can remove lipid peroxides. Animal experiments have shown that riboflavin deficiency is associated with redox system metabolism disorder, lipid peroxide accumulation, decreased GSH activity, increased oxidized glutathione (GSSG) activity, and decreased GSH-PX and GR activity (Horiuchi et al., 1984). Therefore, riboflavin deficiency can contribute to enhanced lipid peroxidation. Moreover, glycine, glutamic acid, and cysteine are the raw materials for the synthesis of GSH. Glycine is essential for the metabolism of glycine, serine, and threonine. The reduction of glycine level indirectly affects the synthesis of GSH and the body’s antioxidant capacity, SGN is capable of restoring the homeostasis of related metabolic pathways in SGU rats, indicating that SGN could treat GU via the inhibition of lipid peroxidation.

### 4.2 Potential Active Components and Mechanism of Action of SGN Against AGU Based on Network Pharmacology

The chemical composition of TCM is intricate and includes many active ingredients. Moreover, the ingredients have different biological activities, physical and chemical properties, and contents. Therefore, identifying the active ingredients in traditional Chinese medicine is one of the key points to clarify the treatment or defense of traditional Chinese medicine. Based on the network pharmacology analysis, a total of 17 active components of SGN were screened out, and the drug-component-target network model was constructed; the key active components were mainly isofraxidin, astilbin, and rosmarinic, caffeic, neochlorogenic, and chlorogenic acid. Previous studies have demonstrated that rosmarinic acid and astilbin exhibit various pharmacological activities, such as anti-inflammatory (Chu et al., 2012), hepatoprotective (Guo et al., 2020), and anti-oxidative activities (Sharma et al., 2020; Wang et al., 2020). Phenolic compounds, including neochlorogenic, chlorogenic, cryptochlorogenic, and caffeic acid, can treat metabolic syndrome since they exert anti-oxidative, anti-inflammatory, and antilipidemic effects (Banjari et al., 2017; Santana-Galvez et al., 2017; Zhao et al., 2020). More importantly, isofraxidin is used as an index component for evaluating the quality of SGN medicinal materials and their preparations in the Chinese Pharmacopoeia; it mainly exhibits anti-inflammatory activity and is a potential ROS scavenger (Li et al., 2014; Su et al., 2019; Liu et al., 2020).

The 28 anti-AGU targets of SGN were found through target mapping. The PPI network revealed that the hub targets mainly include PTGS2, VEGFA, CASP3, IL6, MMP2, MMP9, MAPK1, and KDR and that these targets are closely related to the pathogenesis of AGU. CASP3 is the most important terminal cleavage enzyme in the process of cell apoptosis, whose high expression is usually closely related to the pathogenesis of peptic ulcers (Xie et al., 2020). MMPs play a critical role in the degradation of gastric extracellular matrix proteins. MMP9 is involved in the repair of GU tissue and can also recruit inflammatory cells and participate in the inflammatory response. MMP2 is a gelatinase involved in the remodeling of the extracellular matrix required for the healing process (Rudra et al., 2022). It is well known that VEGF (including VEGFA) is a powerful stimulator of angiogenesis, and it plays a crucial role in angiogenesis and the promotion of wound healing (Bueno et al., 2021). As an endogenous vasodilator factor, NO can regulate gastric acid, gastric mucus secretion, and bicarbonate production and plays a direct role in maintaining mucosal integrity and mucosal defense (Almasaudi et al., 2016). Our study showed that SGN treatment could significantly improve the aberrant NO regulation in rats and maintain it at a normal level. Besides, inflammation is essential in the formation and development of AGU, and acute inflammation can stimulate the upregulation of the transcriptional expression of pro-inflammatory factors (TNF-α, 1L-6, and 1L-1β) (Lin et al., 2014). TNF-α, 1L-6, and 1L-1β are the most common pro-inflammatory factors in the body, which can regulate the expression of apoptosis-related genes (Yang et al., 2019).

### 4.3 Integrated Analysis of Metabolomics and Network Pharmacology

**FIGURE 12:**
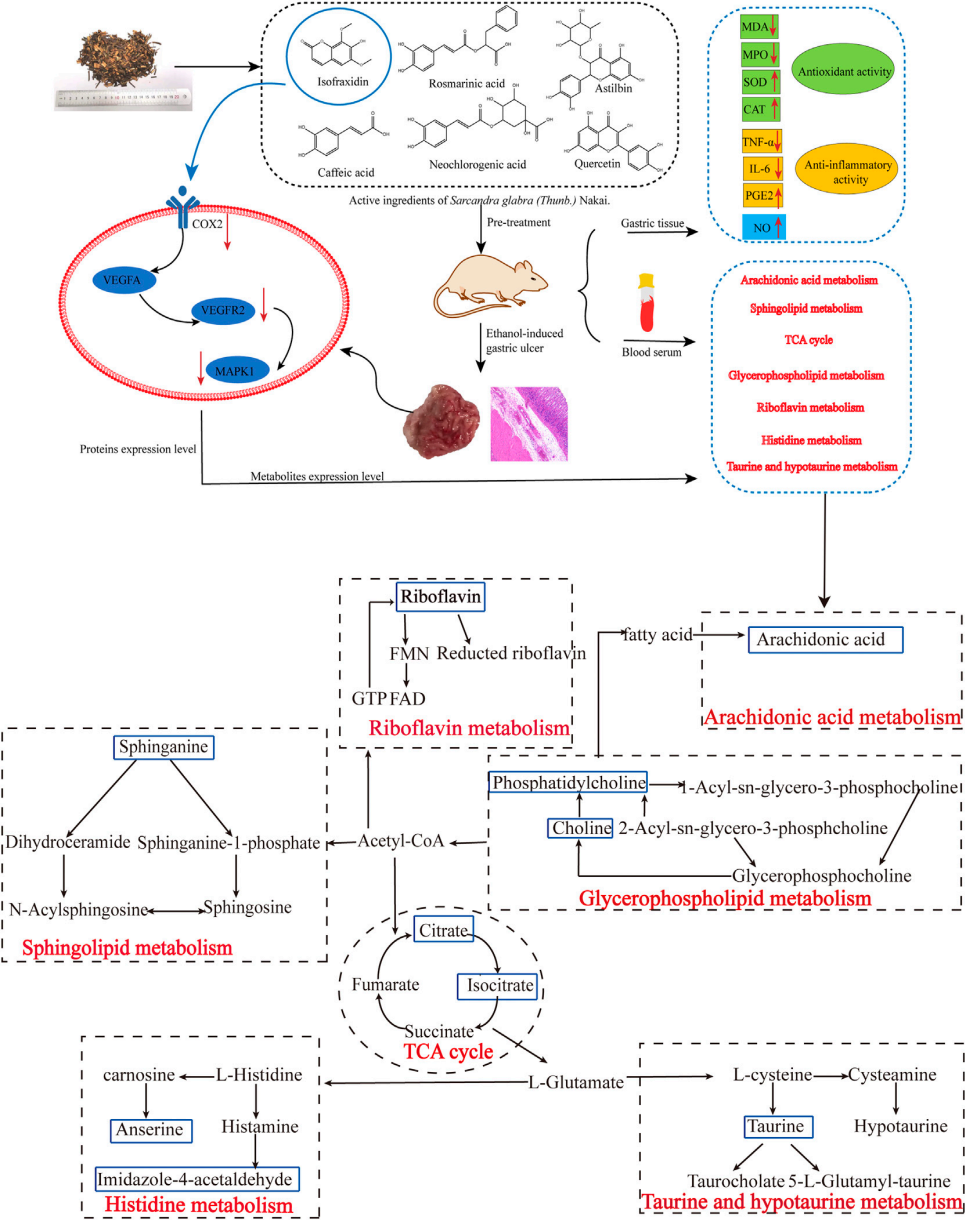
The main mechanisms of SGN against ethanol-induced GU rats based on conjoint analysis of systems biology.

Life activities in cells are joined by genes, proteins, and small molecule metabolites, and the functional changes in upstream (nucleic acid, protein, etc.,) macromolecules are ultimately reflected at the metabolic level, with the metabolome being downstream of gene regulatory networks and protein action networks. Therefore, integrating metabolomics and network pharmacology to analyze the correlation of differential metabolites and potential upstream targets, so as to explore the potential mechanism of SGN anti-AGU, is warranted. The results of the integrated analysis indicated that excessive alcohol could stimulate the microcirculation of blood vessels on the gastric mucosa, producing several inflammatory mediators and cytokines, which disturb the normal structure and physiological function of gastric tissue (Zeng et al., 2017). The inflammation can then stimulate the metabolism of AA to release its metabolites, leading to inflammatory reactions, such as fever, pain, and vasodilation. Prostaglandins (PGs) are the main metabolites of AA. PGE2 has various biological functions and provides local protection for the gastric mucosa. Furthermore, it can increase gastric mucosal blood flow, vascular permeability, and glandular mucus secretion, enhancing the local mucosal barrier function. It can also promote the regeneration of gastric mucosal epithelial cells, induce basal mucosal cells to migrate to the surface, and promote the self-repair of the ulcer surface (Amorim et al., 2016). PGs biosynthesis is regulated by COX-1 and COX-2, also named PTGS2 and PTGS1 (Hirose et al., 2002). PTGS2 is an important enzyme induced in the inflammatory process and traditional anti-GU drugs including NSAIDs are able to exert anti-inflammatory effects by inhibiting COX-2. In addition, previous studies have found that COX-2 is highly expressed in GU tissue. Inhibiting COX-2 can reduce gastric acid secretion in patients with GU, promoting the healing of GU, and reduce recurrence (Saxena et al., 2008; Hu et al., 2020). Furthermore, it can induce tissues to produce VEGFA-KDR, also called VEGFR2, which plays an important role in regulating endothelial cell proliferation and differentiation. Inhibition of VEGFR2 is also an option for gastric cancer targeted therapy. Moreover, it is the main mediator of VEGF-induced angiogenesis signal transduction. After VEGFA binds to its receptor KDR, it activates MAPK1/8 or c-Jun N-terminal kinase (JNK). The activation of MAPK1 is closely related to the invasion of gastric cancer cells, and knockdown of MAPK1 expression in gastric cancer cell lines can inhibit cell proliferation, migration and invasion, and induce apoptosis (Lim et al., 2018; Sierra et al., 2018). Furthermore, MAPK1 is involved in sphingolipid metabolism, and sphingolipid signaling may also be essential in numerous pathophysiologies, such as inflammation, vascular injury, and cancer (Batheja et al., 2003). Even more importantly, based on the algorithms for network pharmacology and analysis results of HPLC, isofraxidin was finally selected as key active ingredient. Additionally, the proteins with high degree value, that also appeared in the integrated analysis, were selected, as the critical proteins and experimental verification was carried out. The results of the western blot analysis indicated that both SGN and isofraxidin could inhibit the abnormal expression of these hub proteins (See Figure 12).

## 5 Conclusion

Taken together, SGN can prevent alcohol-induced AGU most likely by protecting the integrity of the gastric mucosa, improving the tissue oxidative stress state, and inhibiting the expression of inflammatory factors. However, this study has some limitations. 1) The functions of the metabolites identified via metabolomics are unknown in the body and require more in-depth research; 2) Most KEGG pathways were predicted through network pharmacology, and only two metabolic pathways were consistent with the results of metabolomics; 3) The active ingredients in SGN were only identified through HPLC and public databases, which cannot identify components in the blood; and 4) The results acquired from rats may not be directly extrapolated to humans. In the future, a comprehensive analysis of the blood components of SGN will be carried out. Furthermore, the SGN-based anti-GU mechanism is mainly related to oxidative stress. *NFE2L2*, predicted by the network pharmacology, encodes nuclear factor erythroid 2 related factor 2 (Nrf2) protein, and the Kelch-like ECH-associated protein 1 (Keap1)-Nrf2/antioxidant response element (ARE) signaling pathway is the crucial antioxidant pathway in the body. In the future, we will further analyze the deep molecular mechanism of action of SGN against GU in relation to the Keap1-Nrf2/ARE signaling pathway. Ultimately, clinical studies on SGN will be conducted to better understand the gastroprotective efficacy of SGN on the human body.

## Data Availability

The original contributions presented in the study are included in the article/Supplementary Material, further inquiries can be directed to the corresponding authors.

## Abbreviations

AGU, acute gastric ulcer; CASP3, Caspase-3; CAT, catalase; ELISA, enzyme-linked immunosorbent assay; GU, gastric ulcer; HE, Hematoxylin-eosin; IL-1β, Interleukin-1β; KDR, Vascular Endothelial Growth Factor Receptor-2; MAPK1, Mitogen-Activated Protein Kinase 1; NFE2L2, Nuclear factor erythroid 2-related factor 2; PPI, Protein-protein interaction; MDA, Malondialdehyde; MMP2, matrix metalloproteinase-2; MMP9, matrix metalloproteinase-9; MPO, myeloperoxidase; OPLS-DA, Orthogonal Partial Least Squares Discriminant Analysis; PCA, principal components analysis; PGE2, Prostaglandin E2; PTGS2, prostaglandin-endoperoxide synthase 2 / cyclooxygenase-2; SGN, Sarcandra glabra (Thunb.) Nakai; TCM, traditional Chinese medicine; TCMSP, Traditional Chinese Medicine Systems Pharmacology; TNF-α, Tumor Necrosis Factor-α; VEGFA, vascular endothelial growth factor A.
