# Supplementary material for: Assessment of the Potential of Sarcandra glabra (Thunb.) Nakai. in Treating Ethanol-Induced Gastric Ulcer in Rats Based on Metabolomics and Network Analysis
Source: Front Pharmacol. 2022 Jul 12;13:810344. doi: 10.3389/fphar.2022.810344 (PMC9315220; doi:10.3389/fphar.2022.810344)
Supplement: Supplementary file 1 [file DataSheet1.docx]

16

15

17

14

13

12

11

16

15

17

14

13

12

11


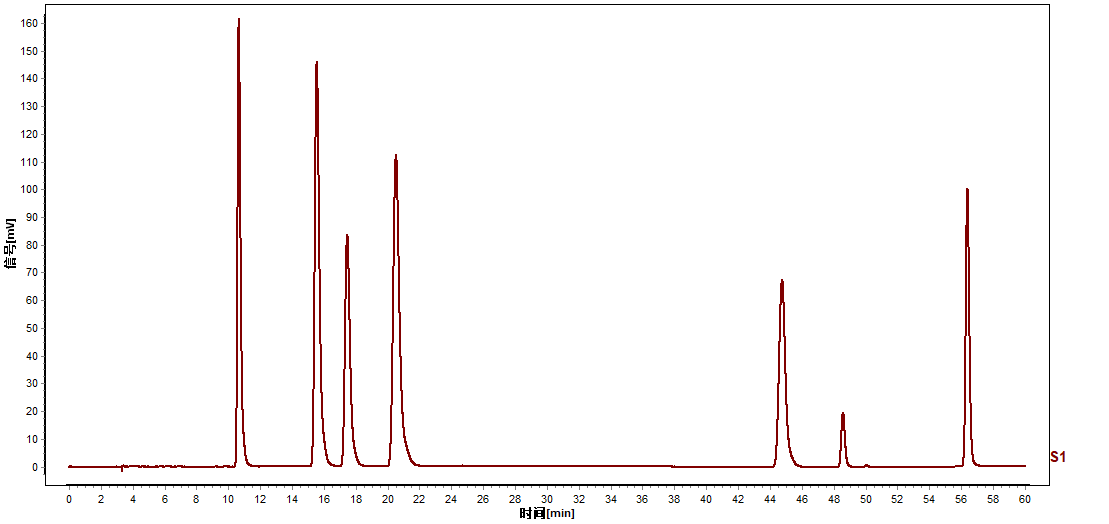

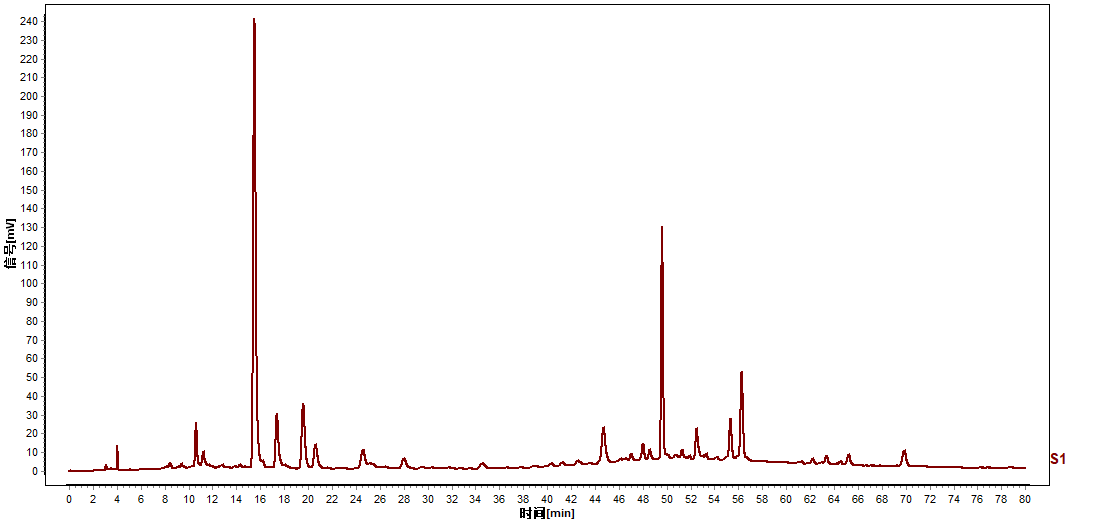


HPLC of standard reference material and SGN decoction at 330nm

HPLC of SGN decoction 330nm. 11- Neochlorogenic acid 12- Chlorogenic acid 13- Cryptochlorogenic acid 14- Caffeic acid 15- Isofraxidin 16- Astilbin 17- Rosmarinic acid

Figure S1 HPLC chromatograms of active ingredients in SGN and corresponding reference substances at different wavelengths


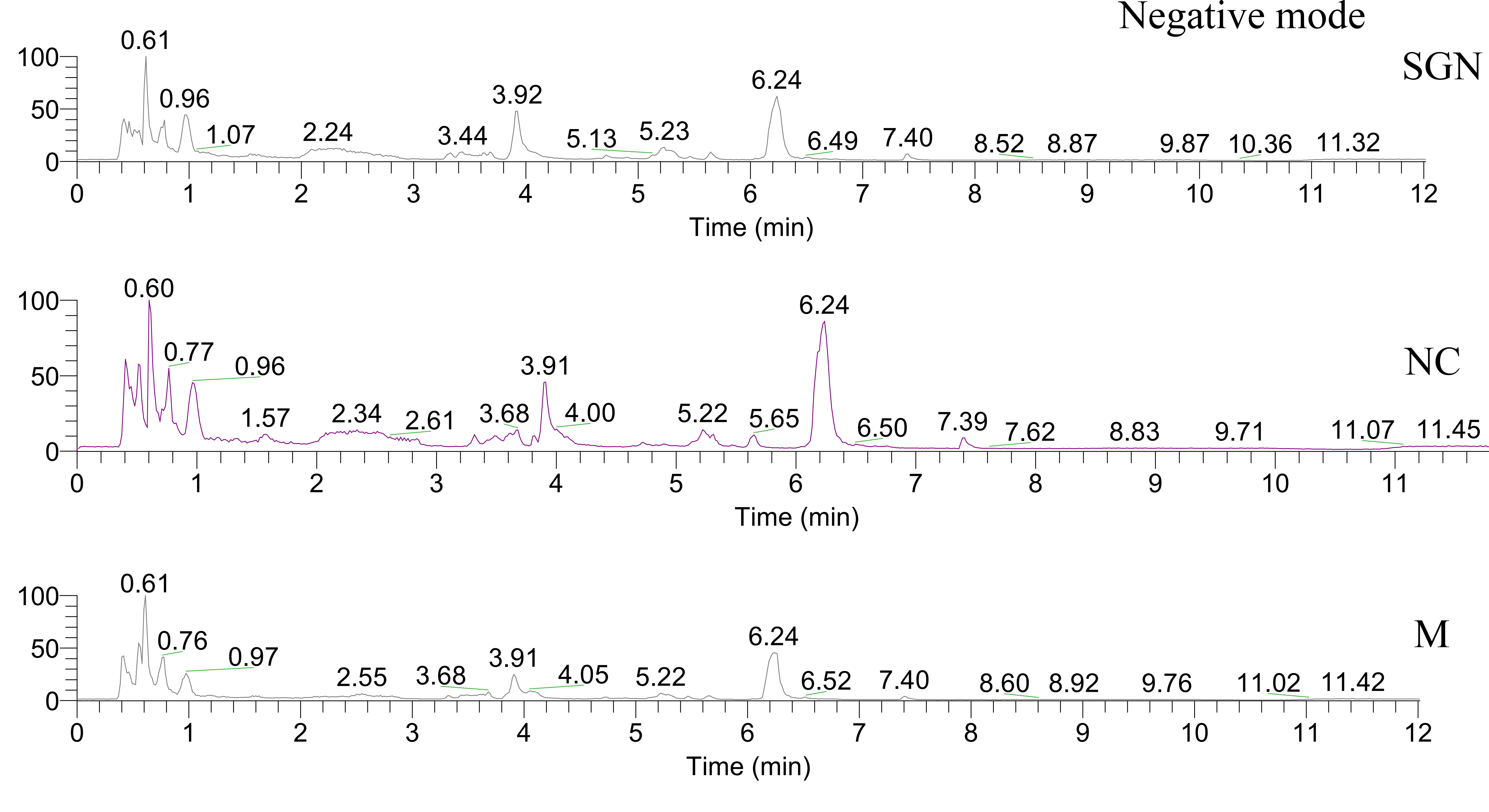

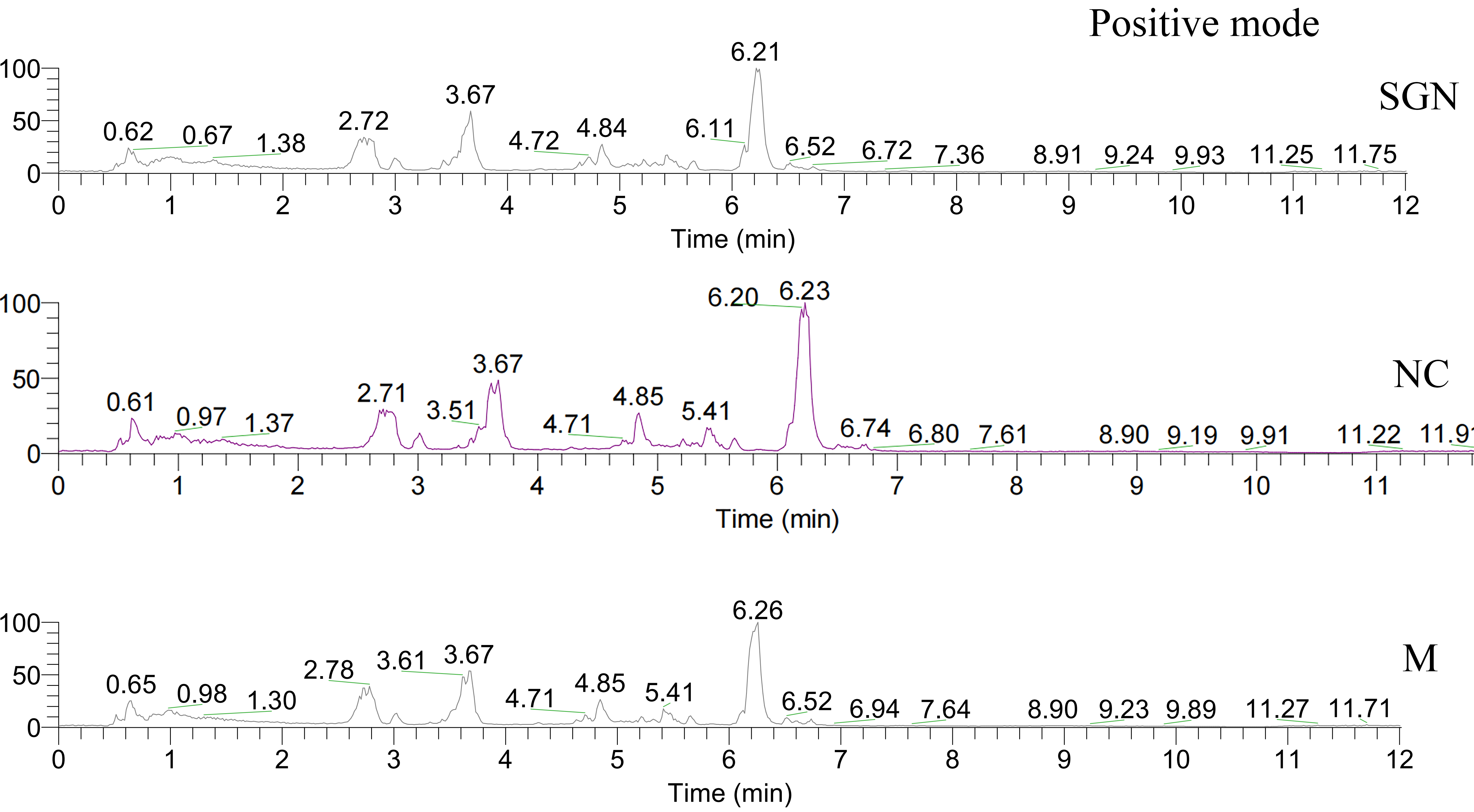


Figure S2 Total ion chromatograms of the M, NC and SGN groups in negative model and positive mode

Table S1 The profile of SGN active ingredient

| NO | Mol ID | Compound | OB (%) | DL | Source |
| --- | --- | --- | --- | --- | --- |
| 1 | MOL000358 | beta-sitosterol | 36.91 | 0.75 | TCMSP |
| 2 | MOL000359 | sitosterol | 36.91 | 0.75 | TCMSP |
| 3 | MOL004373 | Anhydroicaritin | 45.41 | 0.44 | TCMSP |
| 4 | MOL004568 | Engelitin | 36.27 | 0.7 | TCMSP |
| 5 | MOL007132 | (2R)-3-(3,4-dihydroxyphenyl)-2-[(Z)-3-(3,4-dihydroxyphenyl)acryloyl]oxy-propionic acid | 109.38 | 0.35 | TCMSP |
| 6 | MOL007742 | Istanbulin-A | 80.1 | 0.2 | TCMSP |
| 7 | MOL007743 | ZINC00391893 | 41.92 | 0.2 | TCMSP |
| 8 | MOL007744 | CHLORANTHALACTONE A | 41.72 | 0.18 | TCMSP |
| 9 | MOL007747 | chloranoside a_qt | 84.11 | 0.23 | TCMSP |
| 10 | MOL000098 | quercetin | 46.43 | 0.28 | TCMSP |
| 11 |  | Neochlorogenic acid |  |  | HPLC |
| 12 |  | Chlorogenic acid |  |  | HPLC |
| 13 |  | Cryptochlorogenic acid |  |  | HPLC |
| 14 |  | Caffeic acid |  |  | HPLC |
| 15 |  | Isofraxidin |  |  | HPLC |
| 16 |  | Astilbin |  |  | HPLC |
| 17 |  | Rosmarinic acid |  |  | HPLC |


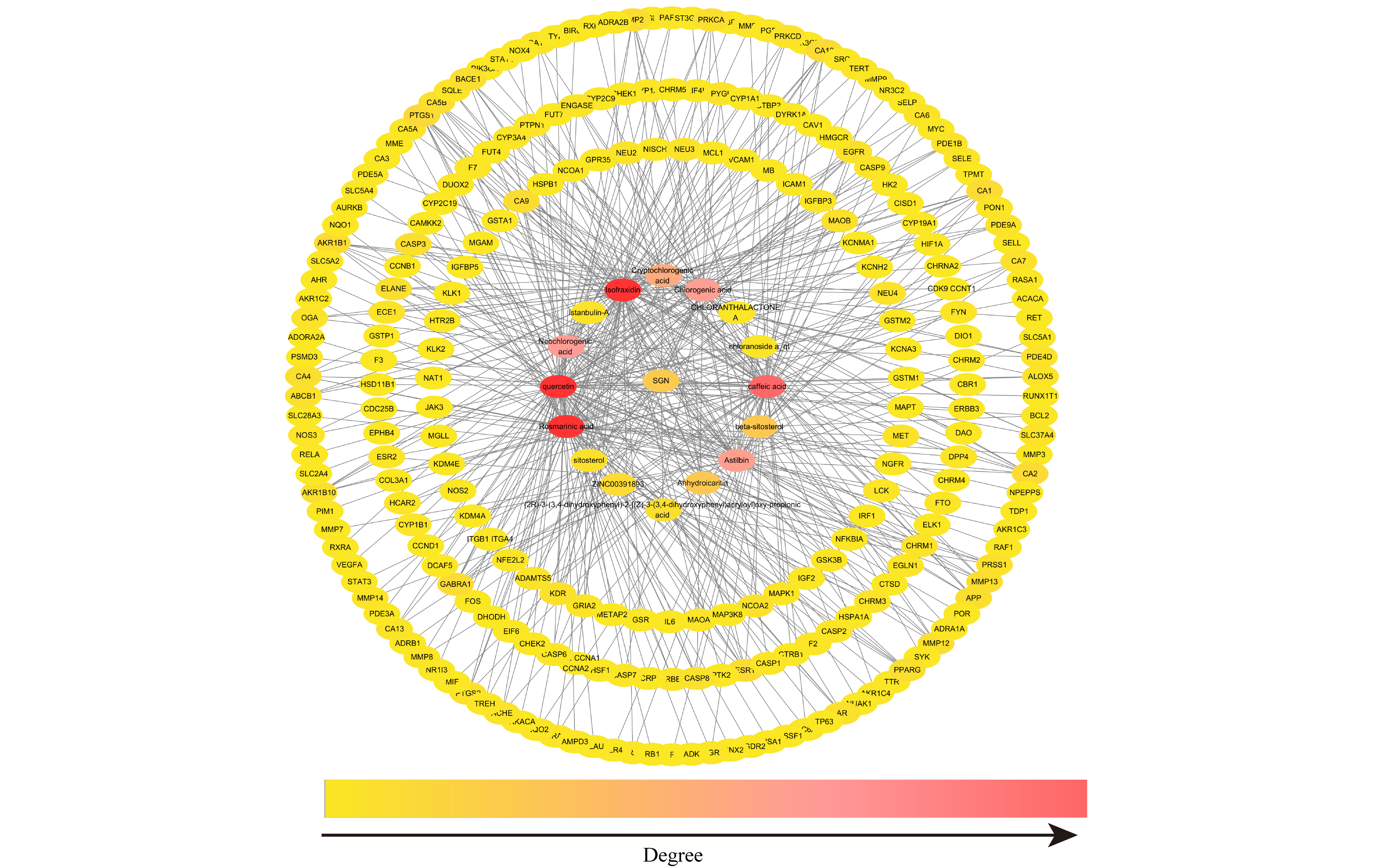


Figure S4 The network of drug- active ingredients-targets

Table S2 Topological analysis of main components in SGN

| Compound | Average Shortest Path Length | Betweenness Centrality | Closeness Centrality | Degree |
| --- | --- | --- | --- | --- |
| quercetin | 2.352490421 | 0.395951981 | 0.425081433 | 78 |
| Isofraxidin | 2.436781609 | 0.279274314 | 0.410377358 | 68 |
| Rosmarinic acid | 2.46743295 | 0.2452047 | 0.405279503 | 63 |
| caffeic acid | 2.482758621 | 0.217849477 | 0.402777778 | 62 |
| Neochlorogenic acid | 2.636015326 | 0.075234293 | 0.379360465 | 40 |
| Chlorogenic acid | 2.643678161 | 0.070844455 | 0.37826087 | 39 |
| Astilbin | 2.651340996 | 0.152608136 | 0.37716763 | 38 |
| Cryptochlorogenic acid | 2.697318008 | 0.053412737 | 0.370738636 | 32 |
| beta-sitosterol | 2.812260536 | 0.045791973 | 0.355585831 | 17 |
| Anhydroicaritin | 2.812260536 | 0.045507569 | 0.355585831 | 17 |
| ZINC00391893 | 2.888889 | 0.007319 | 0.346154 | 7 |
| (2R)-3-(3,4-dihydroxyphenyl)-2-[(Z)-3-(3,4-dihydroxyphenyl)acryloyl]oxy-propionic acid | 2.904215 | 9.68E-04 | 0.344327 | 5 |
| sitosterol | 2.911877 | 0.009908 | 0.343421 | 4 |
| Istanbulin-A | 2.91954 | 9.02E-04 | 0.34252 | 3 |
| CHLORANTHALACTONE A | 2.927203 | 1.27E-04 | 0.341623 | 2 |
| chloranoside a_qt | 2.927203 | 1.27E-04 | 0.341623 | 2 |

Table S5 The key target of SGN to against GA and its topological properties

| Gene | Protein | Average Shortest Path Length | Betweenness Centrality | Closeness Centrality | Degree |
| --- | --- | --- | --- | --- | --- |
| PTGS2 | Prostaglandin-Endoperoxide Synthase 2 | 1.111111111 | 0.178824974 | 0.9 | 24 |
| VEGFA | Vascular Endothelial Growth Factor A | 1.148148148 | 0.101020031 | 0.870968 | 23 |
| IL6 | Interleukin 6 | 1.185185185 | 0.066757348 | 0.84375 | 22 |
| CASP3 | Caspase 3 | 1.296296296 | 0.046841462 | 0.771429 | 19 |
| MAPK1 | Mitogen-Activated Protein Kinase 1 | 1.296296296 | 0.046408356 | 0.771429 | 19 |
| MMP9 | Matrix Metallopeptidase 9 | 1.37037037 | 0.014576473 | 0.72973 | 18 |
| MMP2 | Matrix Metallopeptidase 2 | 1.407407407 | 0.011272986 | 0.710526 | 17 |
| KDR | Vascular endothelial growth factor receptor-2 | 1.444444444 | 0.023906854 | 0.692308 | 16 |
| NOS3 | Nitric Oxide Synthase 3 | 1.444444444 | 0.013493328 | 0.692308 | 15 |
| HIF1A | Hypoxia Inducible Factor 1 Subunit Alpha | 1.444444444 | 0.025284932 | 0.692308 | 15 |
| VCAM1 | Vascular Cell Adhesion Molecule 1 | 1.518518519 | 0.006679432 | 0.658537 | 14 |
| PPARG | Peroxisome Proliferator Activated Receptor Gamma | 1.481481481 | 0.027400306 | 0.675 | 14 |
| MMP3 | Matrix Metallopeptidase 3 | 1.555555556 | 0.002591441 | 0.642857 | 13 |
| PLAU | Plasminogen Activator, Urokinase | 1.555555556 | 0.004448843 | 0.642857 | 13 |
| MET | MET Proto-Oncogene, Receptor Tyrosine Kinase | 1.62962963 | 0.002249808 | 0.613636 | 11 |
| NOS2 | Nitric Oxide Synthase 2 | 1.62962963 | 0.003108003 | 0.613636 | 11 |
| MMP7 | Matrix Metallopeptidase 7 | 1.62962963 | 0.001044634 | 0.613636 | 11 |
| MMP13 | Matrix Metallopeptidase 13 | 1.666666667 | 4.96E-04 | 0.6 | 10 |
| NFE2L2 | Nuclear Factor, Erythroid 2 Like 2 | 1.666666667 | 0.047770795 | 0.6 | 9 |
| F3 | Coagulation Factor III, Tissue Factor | 1.740740741 | 5.54E-04 | 0.574468 | 9 |
| PTGS1 | Prostaglandin-Endoperoxide Synthase 1 | 1.740740741 | 0.01183432 | 0.574468 | 8 |
| TERT | Telomerase Reverse Transcriptase | 1.777777778 | 0 | 0.5625 | 7 |
| CYP2C9 | Cytochrome P450 Family 2 Subfamily C Member 9 | 1.851851852 | 0.014850882 | 0.54 | 5 |
| CYP2C19 | Cytochrome P450 Family 2 Subfamily C Member 19 | 1.925925926 | 0.007178893 | 0.519231 | 4 |
| BCL2 | BCL2 Apoptosis Regulator | 2.148148148 | 0 | 0.465517 | 3 |
| FUT4 | Fucosyltransferase 4 | 2.037037037 | 0 | 0.490909 | 3 |
| GSTM1 | Glutathione S-Transferase Mu 1 | 2.444444444 | 0.002374169 | 0.409091 | 3 |
| MGLL | Monoglyceride Lipase | 2.037037037 | 0 | 0.490909 | 2 |





Figure S5 Overall compound-reaction-enzyme-gene network. Red hexagons, gray diamonds, green rectangles, and purple circles represent active compounds, reactions, proteins and genes, respectively.

2.7.2 Experimental Validation of Integrated Analysis

2.7.2.1 Materials and reagents

ERK2 (GB11370), COX-2 (GB11077-2), VEGFR2 (GB11190), GAPDH (GB15004) and HRP-labeled goat anti-rabbit IgG (GB23303) were acquired from Wuhan Servicebio Technology Co., Ltd..

2.7.2.2 Grouping and handling of rats

Male Sprague-Dawley (weight: 180-200g) rats were obtained from SIPEIFU Biotechnology Co., Ltd. (Beijing, China) (License NO. SCXK 2019-0010). Except for adding the isofraxidin group(25mg·kg^-1^), the rest of the situation is the same as item 2.3.
